# Compositional homogeneity in the pathobiome of a new, slow-spreading coral disease

**DOI:** 10.1186/s40168-019-0759-6

**Published:** 2019-11-22

**Authors:** Michael Sweet, Alfred Burian, James Fifer, Mark Bulling, David Elliott, Laurie Raymundo

**Affiliations:** 1 Aquatic Research Facility, Environmental Sustainability Research Centre, University of Derby, Derby, UK; 2 Marine Laboratory, University of Guam, Mangilao, GU 96923 Guam

**Keywords:** Microbiome, Cyanobacteria, Coral disease, Anna-Karenina, Microbial networks, Beta-diversity

## Abstract

**Background:**

Coral reefs face unprecedented declines in diversity and cover, a development largely attributed to climate change-induced bleaching and subsequent disease outbreaks. Coral-associated microbiomes may strongly influence the fitness of their hosts and alter heat tolerance and disease susceptibility of coral colonies. Here, we describe a new coral disease found in Micronesia and present a detailed assessment of infection-driven changes in the coral microbiome.

**Results:**

Combining field monitoring and histological, microscopic and next-generation barcoding assessments, we demonstrate that the outbreak of the disease, named ‘grey-patch disease’, is associated with the establishment of cyanobacterial biofilm overgrowing coral tissue. The disease is characterised by slow progression rates, with coral tissue sometimes growing back over the GPD biofilm. Network analysis of the corals’ microbiome highlighted the clustering of specific microbes which appeared to benefit from the onset of disease, resulting in the formation of ‘infection clusters’ in the microbiomes of apparently healthy corals.

**Conclusions:**

Our results appear to be in contrast to the recently proposed Anna-Karenina principle, which states that disturbances (such as disease) trigger chaotic dynamics in microbial communities and increase β-diversity. Here, we show significantly higher community similarity (compositional homogeneity) in the pathobiome of diseased corals, compared to the microbiome associated with apparently healthy tissue. A possible explanation for this pattern is strong competition between the pathogenic community and those associated with the ‘healthy’ coral holobiont, homogenising the composition of the pathobiome. Further, one of our key findings is that multiple agents appear to be involved in degrading the corals’ defences causing the onset of this disease. This supports recent findings indicating a need for a shift from the one-pathogen-one-disease paradigm to exploring the importance of multiple pathogenic players in any given disease.

## Background

Coral communities are characterised by high species and genetic diversity, and both are currently threatened by a number of environmental stressors [1]. In 2017, reefs (on a global scale) experienced an extended period of heat stress, which led to the third global bleaching event since the start of records in the 1980s [2]. During this pan-tropical episode of coral bleaching, locations known for their high coral cover and diversity such as the Great Barrier Reef and the Maldives archipelago were affected by large scale coral die-offs [2, 3]. The causal links between climate change (i.e. increased intensity and frequency of heat stress) and coral bleaching are widely considered as drivers of reduced coral cover and loss of functional complexity [4, 5]. However, the few studies which have monitored mass die-offs (over sufficient time periods) suggest that reef degradation might be associated with multiple causal agents. For example, a tagging study in the northeast Caribbean revealed that coral disease infections and coral bleaching might be strongly interactive [6]. While 90% of coral colonies monitored in this study displayed physical signs of thermal stress, many colonies started to show signs of recovery when a disease outbreak radically reduced coral cover, months after the bleaching event [6].

The potential ecological importance of coral diseases has also been emphasised by Maynard et al. [7] who argue that the effect of climate change on coral communities by promoting disease outbreaks may be equally strong or even stronger than its impact via increased bleaching stress. Nevertheless, many coral diseases remain insufficiently characterised and little is known about their aetiologies, transmission dynamics and infection risks, under different climate scenarios [8]. Indeed, for many diseases, even the identity of causal agents is in question [9], leading to uncertainty about the total number of described diseases [10].

A major issue complicating disease characterisation is the complexity of microbial communities associated with coral colonies [11]. Scleractinian corals host a large diversity of bacteria, archaea and fungi [12, 13], which appear compartmentalised within the coral holobiont [14]. It is well known that disease outbreaks induce profound structural changes in these coral-associated microbiomes [15]. Potential pathogenic agents, for example, may actively alter the composition of healthy microbial communities and thereby increase their virulence [16–18]. Further, diseases are not necessarily caused by a single pathogen. Instead, research on black band disease (BBD), the first systematically described coral disease, has clearly demonstrated that disease outbreaks can be linked to several interacting causal agents [15, 19, 20].

Recently, a close relationship between the heat tolerance of coral colonies and the composition of their microbiomes has been demonstrated, linking microbial community organisation to coral functionality [21]. The causal link between heat-stress, disease susceptibility [22] and large shifts in microbiomes following disease infections [15] suggests that microbial community structures may also affect disease resistance in corals [23]. Therefore, the assessment of microbial community structures and the systematic investigation of shifts in the microbiome after disease infections offer a promising pathway to improve our understanding of coral disease dynamics and virulence.

A recent hypothesis that describes the composition of microbial communities in stressed animal hosts is the Anna-Karenina principle [24]. It states that stress and disturbances lead to a decrease of community similarity, paralleling Leo Tolstoy’s dictum that ‘all happy families look alike; each unhappy family is unhappy in its own way’ [25]. The proposed mechanism behind the Anna-Karenina principle (in corals) is that the host organises its microbiome to support beneficial microbes and the resulting selection pressure synchronises microbial communities. Indeed, it has recently been shown that local host identity plays a dominant role in structuring the microbiome [26], and despite high levels of microbial diversity, key signature members of the microbiome (presumably those beneficial to the host) are stable in both space and time [26], at least for some species. However, that said, if communities become disturbed, opportunistic microbes will start to dominate and stochastic processes should (at least in theory) lead to decreased community similarity [24].

Here, we describe a newly identified coral disease named ‘grey-patch disease’ and assess the changes in microbial community structure associated with its outbreak. Our main research hypotheses were (1) several interacting pathogens weaken the coral host to facilitate an outbreak of grey-patch disease and (2) infection results in divergence of microbial communities between colonies, in accordance with the Anna-Karenina principle. In order to address these hypotheses, we combined microscopic and histological approaches with field surveys and microbial analyses—which also allowed us to simultaneously achieve a detailed characterisation of this new coral disease.

## Results

**Fig. 1:**
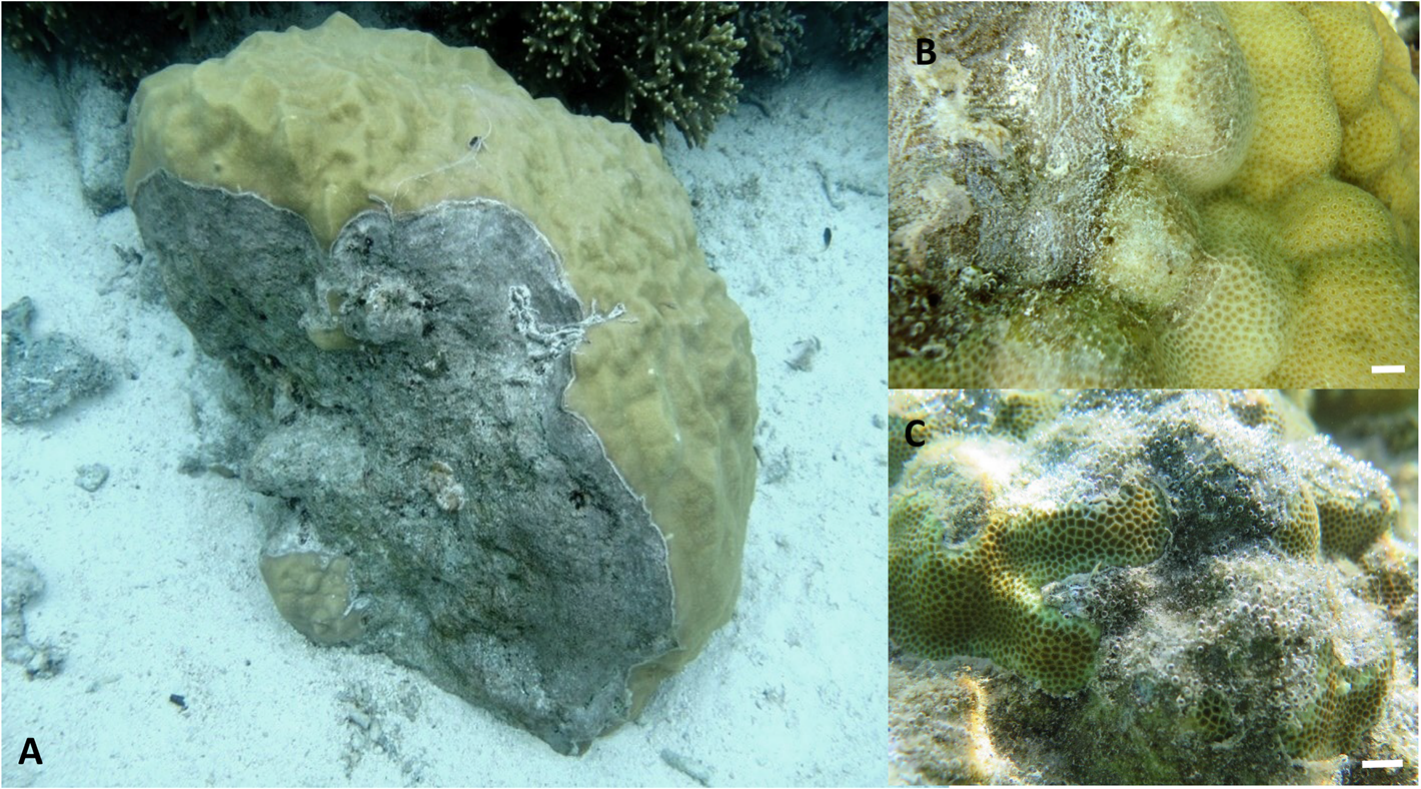
Macroscopic signs of grey-patch disease. Large patches were associated with some colonies (**a**). **b**, **c** Higher resolution images of the disease lesions with coral tissue paling at the lesion interface (**b**). Characteristic bubbles associated with active lesions can be observed in **b** and **c**. Scale bars for **b** and **c** indicate 5 mm

Grey-patch disease (GPD) is macroscopically detectable by the presence of varying-sized mats of thin, grey-coloured biofilms occurring at multiple locations on the same colony (i.e. multifocal lesions), which do not appear to be limited to a particular location on a colony (Fig. 1a and Additional file 1: Figure S1). Eighteen different species of coral, from 10 distinct genera including *Porites*, *Acropora* and *Montipora* showed signs of infection by GPD (see Additional file 1: Table S1 for a full list and Additional file 1: Figure S2 for examples of GPD on different coral genera)*.* The grey biofilms on diseased colonies ranged from coin-sized areas (5 cm^2^) to over 1 m^2^ patches in some of the larger infected *Porites* colonies. In this genus, infected colonies were often misshapen, as colonies had continued to grow around the affected areas. In all species, the grey biofilm closely adhered to the coral skeleton and lesions had a discrete, delineated border adjacent to apparently healthy coral tissue. Various ‘interaction states’ were observed during field monitoring (between the coral and the biofilm). We classified lesions as ‘active’ when they exhibited paling colouration of the coral tissue (Fig. 1b) and increased mucous secretion. A further indicator was the presence of small bubbles emerging from the biofilm (Fig. 1b, c; Additional file 1: Figure S1D), suggesting high levels of primary production are occurring at the disease lesion. Lesions, which were ‘re-sheeted’ by healthy tissue, were classified as being in a ‘recovery’ stage. ‘Static’ interactions were defined as lesions that showed neither progression nor re-sheeting.

GPD was only observed within Micronesia, despite considerable survey effort in other regions. In Micronesia, outbreaks could be epidemic, affecting 21.7% of 53 surveyed *Porites* colonies for example in the case of Luminao reef, Guam. Mean prevalence, however, was much lower and ranged between 0.3 and 2.7% in monitored reefs of Guam (flat reefs only), Rota, Tinian, Saipan and Chuuk. In other locations (Philippines, Palau, Maldives and Reunion), no signs of infection were detected.

In 2016, we tagged 12 colonies of massive *Porites* at Luminao reef, Guam, and monitored lesion progression. Eleven out of the 12 exhibited an extension of the GPD, although progression rates were highly variable (1.9–440.0 mm year^−1^) with a mean of 65.4 mm year^−1^. Contrastingly, in 2017, five of the 12 tagged colonies showed ‘re-sheeting’ of the tissue, with coral tissue growing back over the GPD biofilm at a rate of 3.95 (± 2.1) mm year^−1^ and one colony was classified as ‘static’ (see Additional file 1: Figure S3 and Table S2 for details on continuity of disease activity).

**Fig. 2:**
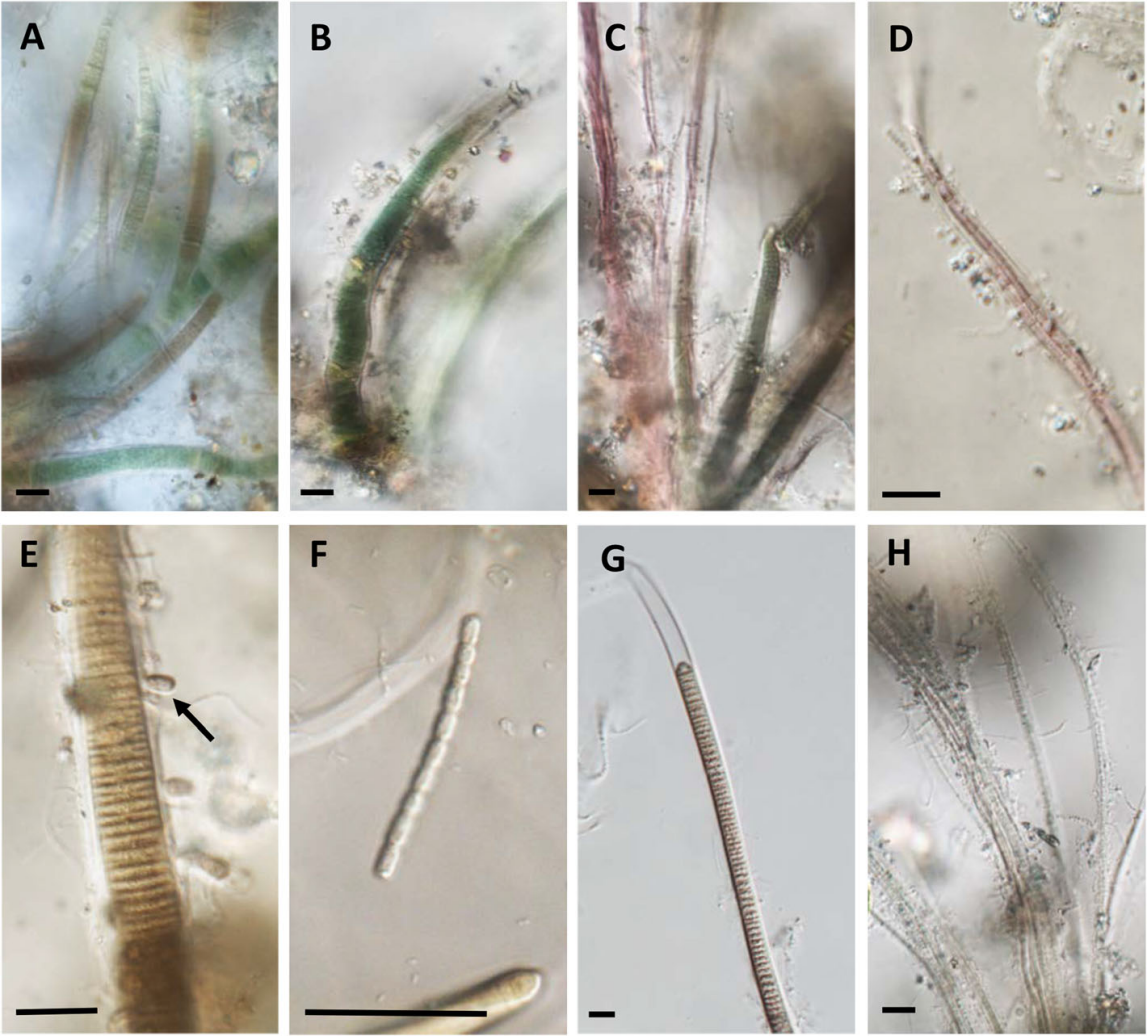
Representative light micrographs of the dominant cyanobacterium present in the GPD biofilm. **a**–**c** Two of the most common cyanobacteria routinely found, morphologically identified as Rivulariaceae. **c**, **d** A representative of *Symploca* with its characteristic pink colouration. **e** The spore forming epithetic cyanobacteria *Entophysalis*. **f** An unidentified but fast free-moving bacterium. **g**, **h** *Phormidium* and another Osciallatoriacae, which both constituted dominant members of the GPD biofilm. Scale bars indicate 10 μm

**Fig. 3:**
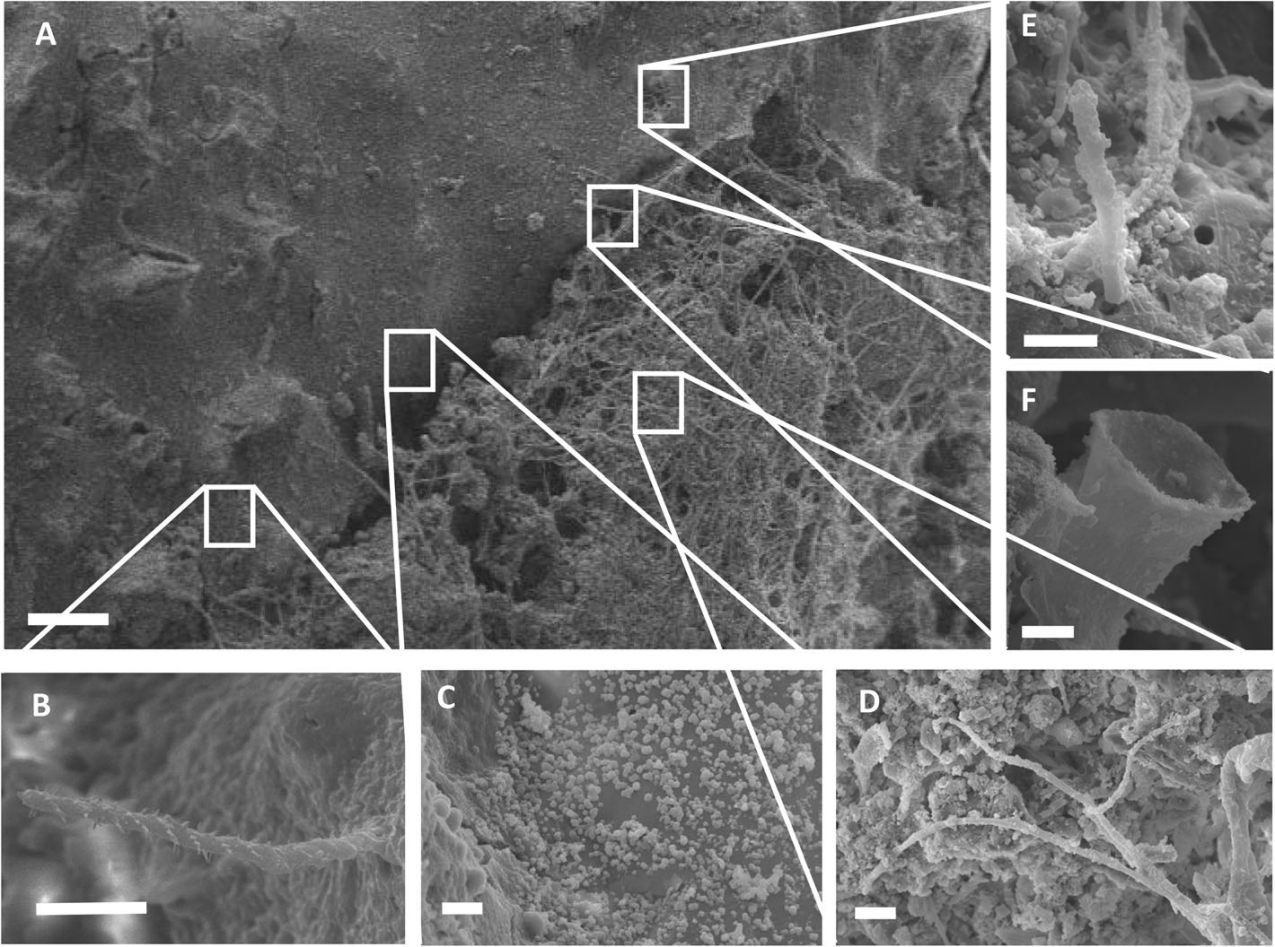
Scanning electron micrographs of corals showing signs of grey-patch disease. **a** The disease-lesion interface. **b**–**f** Common microorgansims associated with the apparently healthy tissue in advance of the lesion interface and/or those present in the advancing GPD biofilm. **c** Large aggregations of coccoid bacteria at the lesion interface. **d** A representative of the fungal hyphae. **e** Cyanobacteria penetrating into coral tissue in advance of the disease lesion. **f** Folliculinid ciliates chitinous lorica, present throughout the lesion interface. Scale bars are **a** 200 μm and **b**–**f** 10 μm

Light micrographs revealed the dominance of several cyanobacteria taxa on diseased colonies (Fig. 2). Two dominant cyanobacteria belonged to the family of Rivulariaceae (Fig. 2a–c), while other commonly encountered taxa included *Symploca* (Fig. 2c, d), the spore-forming epithetic cyanobacterium *Entophysalis* (Fig. 2e), an unidentified and rapidly swimming bacterium (Fig. 2f), *Lyngbia* (Fig. 2e, g) and several *Phormidium* species (Fig. 2h). SEM further confirmed the dominance of cyanobacteria on diseased colonies (Fig. 3b–f) and highlighted physical signs of filaments boring into coral tissue and penetrating the calciodermis (Fig. 3d, e). However, a number of other taxa also seemed to be active at the disease lesion interface. Large aggregations of a coccoid bacterium were routinely found on the surface of otherwise apparently healthy coral tissue, in advance of the lesion interface (Fig. 3c), and folliculinid ciliates (characterised by the chitinous lorica) were also routinely present (Fig. 3f).

**Fig. 4:**
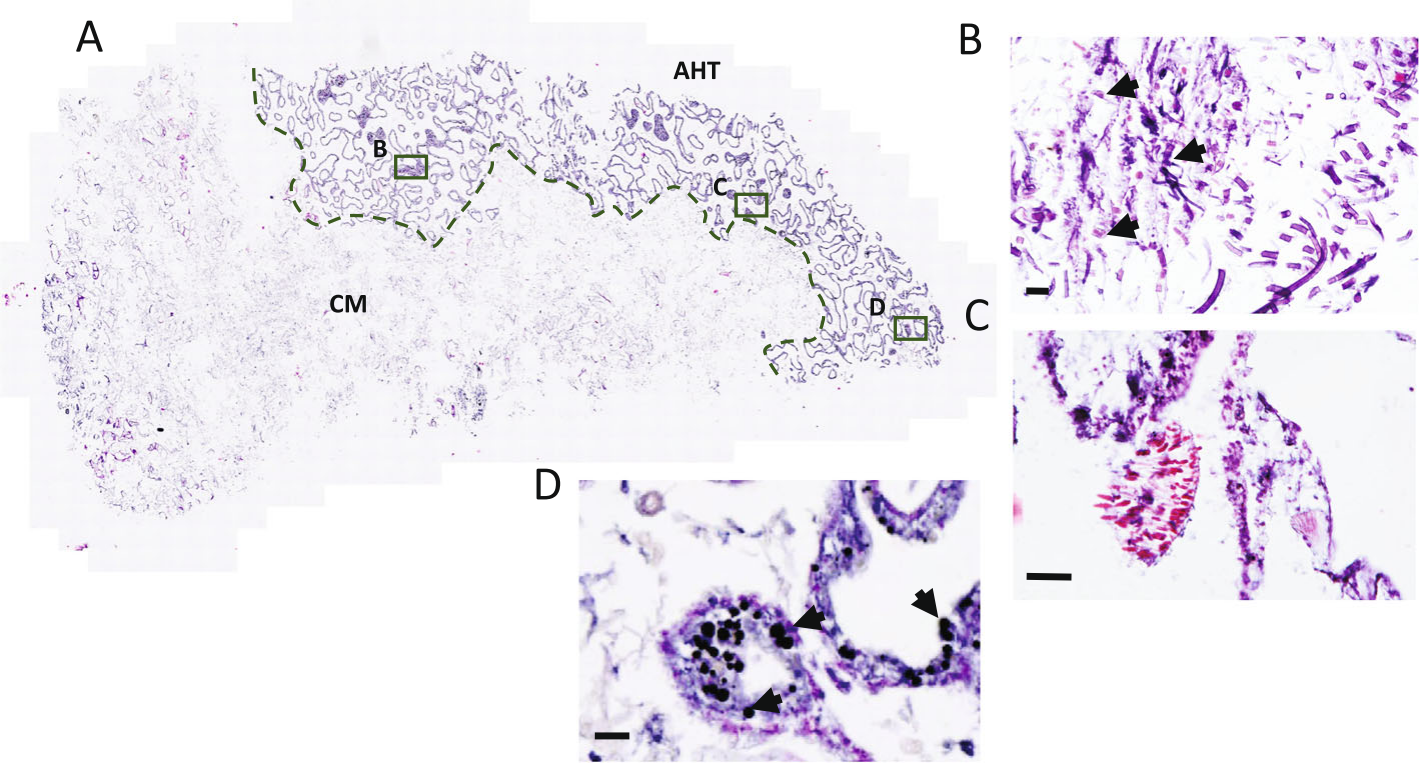
Histological cross-section (**a**) highlighting the sharp demarcation between apparently healthy tissue (AHT), the GPD lesion interface (dashed green line) and the cyanobacterial mat (CM) or biofilm. Rectangles depict positions of panels (**b**–**d**). **b** Arrows highlight cyanobacteria penetrating into the otherwise apparently healthy tissue. **c** Fungal hyphae regularly observed at the lesion interface. **d** Arrows indicate necrotic Symbiodiniaceae at the disease lesion interface. Scale bars are 25 μm

Histological sections revealed a clear demarcation between apparently healthy tissue and the disease biofilm resulting in a clear lesion interface (Fig. 4a). However, both cyanobacteria and fungi actively penetrated this boundary and affected otherwise healthy tissues (Fig. 4b, c). The proposed pathogens progressed furthest in close proximity to the coral calicodermis (visual observations during decalcification and before histological processing), with cyanobacteria (Fig. 4b) and necrotic Symbiodiniaceae cells (Fig. 4d) clearly present in light micrographs. Together, these independent lines of evidence provide indication of infection dynamics preceding macroscopic signs of the disease.

High-throughput 16S prokaryotic analyses of the coral microbiome demonstrated a high diversity of amplicon sequence variants (ASVs; i. e. unique sequences indicating distinct taxa) associated with apparently healthy and diseased corals (8196 unique ASVs). Species richness (per sample) showed significant differences between groups (ANOVA, *F*_(2,24)_ = 27, *p* < 0.001) and was almost four times higher in diseased tissue (1295 ± 400 for diseased coral and 333 ± 231 for apparently healthy coral—see Additional file 1: Table S3). However, diseased tissue had a much higher proportion of very rare taxa than those associated with apparently healthy tissues, although this had only a minor influence on α-diversity and resulted in no significant differences in the other diversity measurements. Shannon’s diversity for diseased tissues was 4.5 (± 0.8) vs 3.5 (± 1.1) for healthy, Simpson’s index was 0.88 (± 0.11) for diseased vs 0.83 (± 0.21) for healthy and Pielou’s evenness scores were 0.63 (± 0.10) and 0.63 (± 0.23) for diseased and healthy tissues, respectively.

ITS analyses revealed the presence of two fungal ribotypes in all infected tissue samples (GenBank Accession Numbers MN306004 and MN306005) and their absence in apparently healthy corals. The two ribotypes had 100% similarity with fungi cultured from corals and were identified (based on unpublished GenBank data) as the genera *Cladosporium* and *Fusarium*. However, we want to note that currently available fungal primers mismatch with coral DNA, and therefore, our approach most likely only detects the dominant taxa in any given sample.

**Fig. 5:**
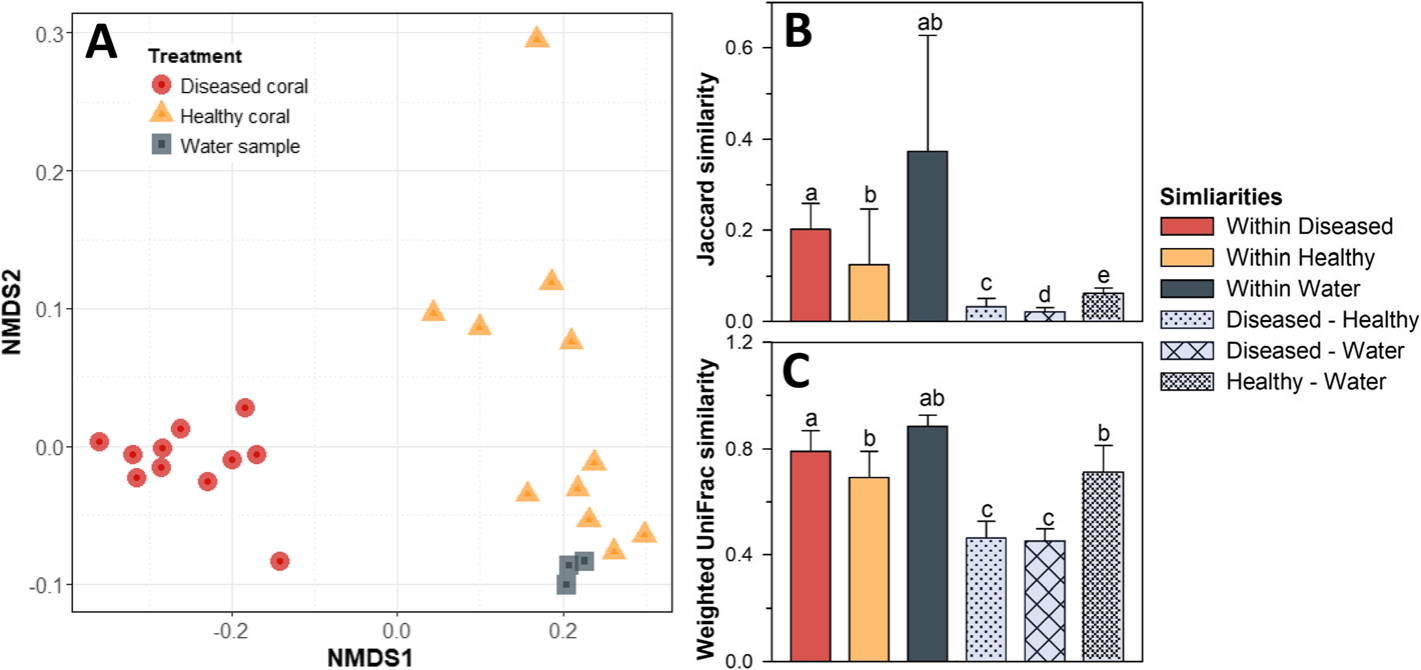
Difference in microbial community structure associated with apparently healthy coral tissue, diseased tissue and water samples. **a** A NMDS plot (stress < 0.1) visualises differences between different sample groups based on weighted UniFrac similarity. **b**–**c** Average similarity of communities within (coloured bars) and between sample groups (grey bars) based on Jaccard and weighted UniFrac similarity. Letters denote significant differences based on Kruskal-Wallis tests and subsequent pairwise comparisons

Analyses of the microbial community composition confirmed the large shifts in community structure (Fig. 5). The ASVs making up the microbial communities of diseased and apparently healthy tissue (8348 unique ASVs found across all samples) significantly differed in their identity and relative frequency (PERMANOVA for both Jaccard and weighted UniFrac distances, *F*_(2,24)_ = 21, *p* < 0.01). Furthermore, the prokaryotic microbial communities in the water samples were significantly different from those associated with diseased (*p* < 0.01) and apparently healthy coral tissue (*p* = 0.015). Despite higher species richness and the destruction of the organised coral tissue (evident by histology), pathobiomes showed a higher within-group similarity than the microbiomes of apparently healthy corals (Fig. 5). Analysis of both, Jaccard and weighted UniFrac indices, demonstrated significantly lower between-community variation among infected corals than among apparently healthy coral (Kruskal-Wallis test *H*_(2)_ > 190, *p* < 0.001; pairwise Wilcoxon test, *p* < 0.001). Between-group comparisons had a strong tendency to have lower similarity than within-group comparisons (Fig. 5b, c), reflecting a high group consistency.

**Fig. 6:**
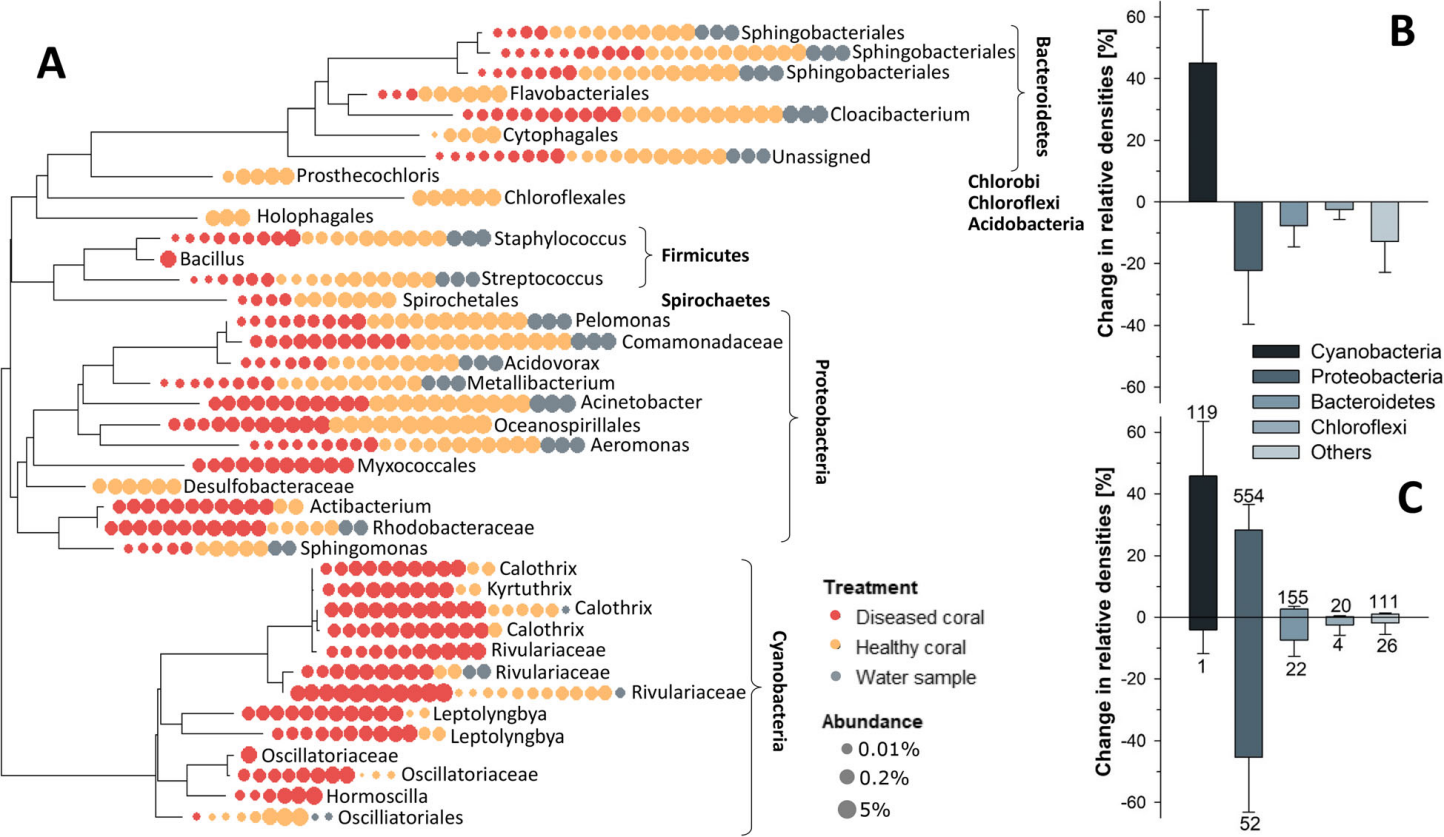
Phylogenetic relationships between ASVs contributing on average > 0.25% to total sequence across all samples (**a**). Dots represent single samples and were colour-coded according to their group affiliation. Dot size represents the relative frequency of ASV in samples of apparently healthy and diseased corals. ASV labels represent lowest taxonomic resolution assigned through the Greengene library algorithm. **b** Comparisons between the relative phyla abundances of microbiomes associated with apparently healthy and diseased coral tissue. Positive and negative values reflect a higher contribution to total number of sequence reads per sample in diseased and healthy coral tissues, respectively. **c** Per phyla sums of ASVs that show significant differences between apparently healthy and diseased tissue. *X*-axis represents the sum of relative frequency changes after disease infection. Numbers above error bars indicate the number of ASVs that show significant differences between groups

A detailed taxonomic analysis of abundant taxa in next-generation sequencing analyses (> 0.25% relative abundance) confirmed our previous observation of a cyanobacteria biofilm forming at the lesion interface and corroborated the histological evidence which illustrated that these cyanobacteria penetrated otherwise healthy tissue in advance of the biofilm (Fig. 4). Multiple cyanobacterial families were present at the lesion interface (evident via sequencing and the light micrographs—Fig. 2), but half of the 14 dominant cyanobacterial ASVs obtained in sequencing data belonged to only 1 family (Rivulariaceae; Fig. 6). Interestingly, many of these disease-dominant cyanobacteria were also identifiable in apparently healthy coral tissue (evident by sequence data), but these occurred at lower relative frequencies. At phylum level, cyanobacteria were the only group that showed a net increase in relative frequency in diseased tissues (from 5 to 50% of total rDNA copies; Fig. 6b). An analysis of all ASVs that differed significantly between sample groups revealed that all dominant phyla contained individual ASVs that profited from disease infections. In fact, the number of ASVs that showed relative increases in diseased tissues was clearly higher in all phyla, although their contribution to total copy numbers was often small (Fig. 6c). Notably, the majority of disease-associated taxa were not present in water samples, whereas dominant ASVs that showed higher densities in apparently healthy tissue were also abundant in the water column (Fig. 6a).

**Fig. 7:**
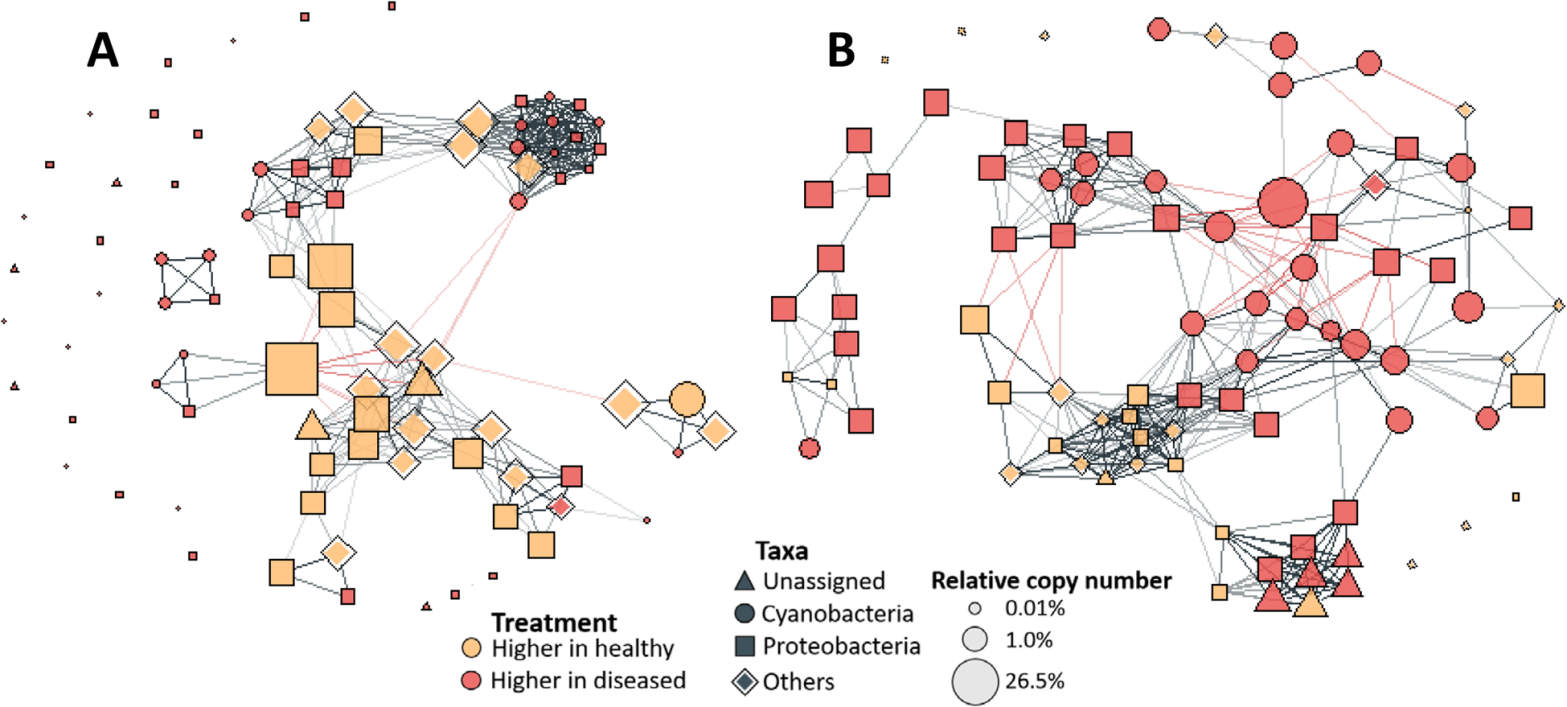
Interactions between dominant ASVs in the microbiome of apparently healthy (**a**) and diseased coral tissues (**b**) depicted in a network representation. Lines reflect significantly positive (black) and negative (red) interactions. Line thickness symbolises the strength of interactions. Yellow and red fills indicate ASVs that show significantly higher densities in apparently healthy and diseased coral tissue, respectively. Phyla association of ASVs is demonstrated by different shapes. Relative size of shapes reflects their average relative contribution to total sequence reads in the sample group. See Additional file 1: Figure S3 for detailed taxonomic information of ASVs included here

Patterns of taxa association in prokaryotic microbial communities were assessed in separate network analyses for apparently healthy and diseased tissues, respectively (Fig. 7). Both networks contained significantly higher numbers of positive than negative connections, and the strengths of positive connections were significantly higher (Kruskal-Wallis tests, *H*_(2)_ > 80, *p* < 0.001). All dominant ASVs (included were ASVs contributing > 0.125% to total reads across all sample) showed significant differences in relative frequencies between apparently healthy and infected coral samples, creating two groups of taxa.

**Fig. 8:**
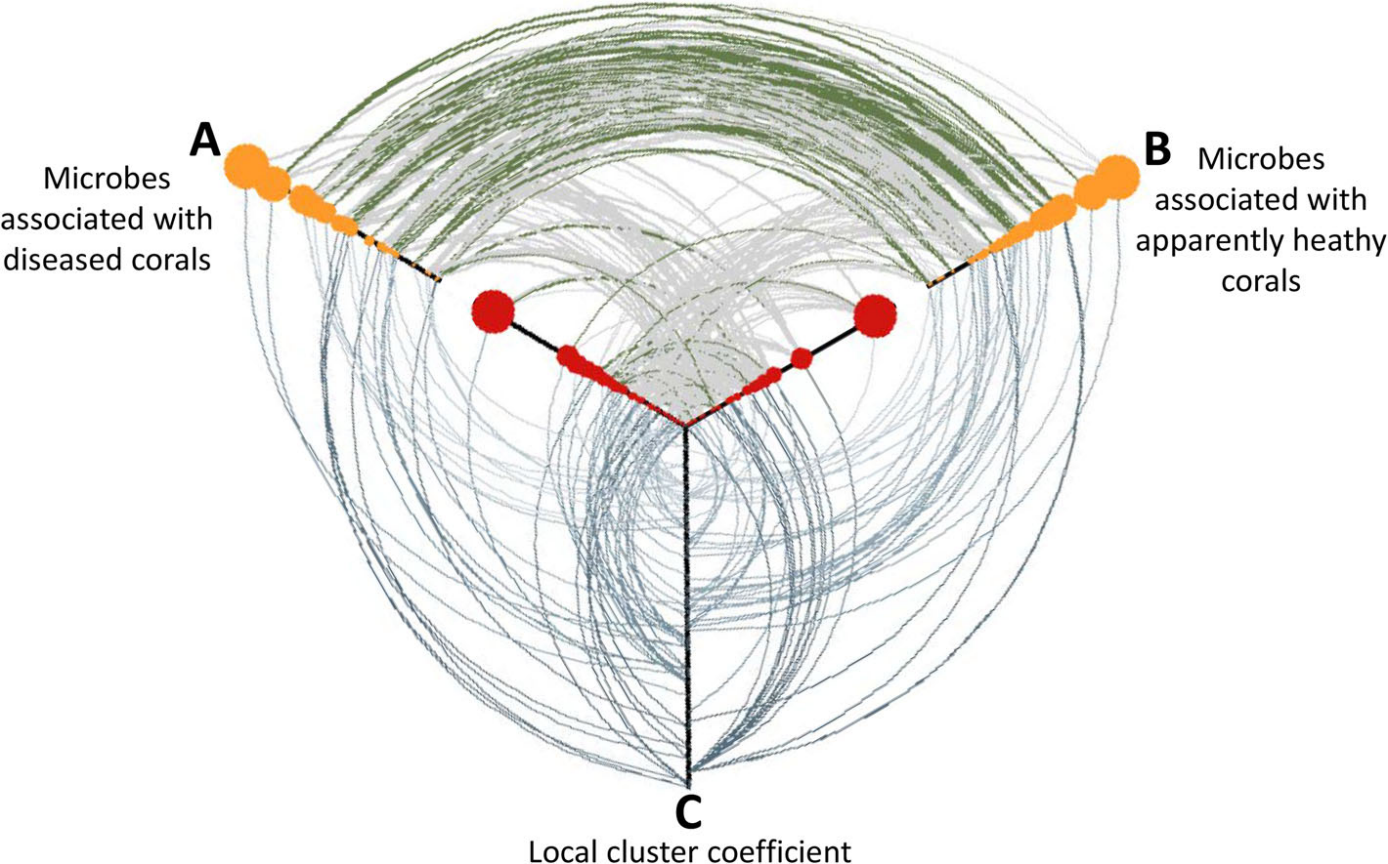
Hive plot assessing characteristics of microbial networks associated with apparently healthy and diseased corals. Displayed are three axes. Points on axes A and B represent all dominant microbes (> 0.125% mean relative abundance across all samples) present in pathobiomes of diseased corals (A) and in the microbiome of apparently healthy corals (B). Both axes are further separated into two sub-axes, one illustrates those microbes with higher densities in apparently healthy tissues (yellow points) and the other illustrates microbes with higher densities in diseased tissues (red points). Size and relative distance from the origin of sub-axis reflect the relative abundance of microbes across all samples. Connections between axes A and B indicate a connection between microbes in both networks of Fig. 7 (green; consistent connection) or in only one of the two networks (grey, i.e. representing an inconsistent connection). Our analysis reveals that consistent connections are mostly found between microbes showing higher relative abundances in apparently healthy corals and that consistency in interactions are rare between microbes benefiting from the diseased state. Axis C illustrates the local clustering coefficient (see the ‘Methods’ section for computation) of microbes in networks of apparently healthy and infected corals. Each microbe on axes A and B is associated with a certain clustering coefficient via a connection to axis C. The distance from the origin of axis C thereby indicates the strength of clustering. Here, microbes, which have higher densities in infected corals, are strongly clustered in the networks of apparently healthy tissues. In the diseased tissue, both ‘groups’ of microbes include a mix of members which are strongly and rather loosely clustered

These two taxa groups showed distinct co-occurrence patterns in community networks. ASVs that were more abundant in diseased tissue formed ‘clusters’ of positively co-occurring ASVs in community networks of apparently healthy tissues (Fig. 7). The same occurred in diseased corals (but to a lesser degree), in that clusters of ASVs, more abundant in apparently healthy tissue were evident. The existence of these clusters (which we refer to as ‘infection’ and ‘survival’ clusters, respectively) was confirmed by in-depth analyses of network structure (Fig. 8). First, we calculated the local cluster coefficients for each ASV, which confirmed that some ASVs benefited from disease infections and clumped together in the networks of apparently healthy corals. We were then able to illustrate that the ASVs within these clusters had a significantly higher saturation of connections, a higher average connection strength and a larger proportion of positive connections with ASVs of the same group, than with ASVs of the opposite group (Kruskal-Wallis tests, *H*_(2)_ > 9, *p* < 0.01; Table 1).

**Table 1:** Descriptive statistics of networks representing interactions between dominant ASV (mean contribution of > 0.125% total sequence reads per sample) in microbiomes of healthy and diseased corals. The rows ‘+ healthy’ and ‘+ diseased’ represent sub-summaries for microbes showing significantly higher relative densities in healthy and diseased samples, respectively. Mean degree indicates the average number of interactions per ASV. Saturation is the number of realised interactions relative to the number of possible interactions. Connection strength refers to mean correlation coefficients between interacting ASVs

Network	No. of species	Mean degree		Saturation all nodes	Saturation within groups	Saturation between groups	Connection strength positive		Connection strength negative		Proportion positive connections	Connection strength within groups		Connection strength between groups	
Healthy	62	10.0	(± 5.0)	16.3	23.1	9.8	0.86	(± 0.09)	0.67	(± 0.04)	0.97	0.87	(± 0.10)	0.83	(± 0.10)
+ Healthy	31	10.1	(± 4.6)	16.6	23.7	9.8	0.81	(± 0.07)	0.68	(± 0.04)	0.94	0.80	(± 0.07)	0.81	(± 0.11)
+ Diseased	31	9.8	(± 5.5)	16.1	22.6	9.8	0.91	(± 0.08)	0.64	–	0.99	0.94	(± 0.08)	0.84	(± 0.08)
Diseased	81	8.7	(± 4.7)	10.9	16.5	6.4	0.77	(± 0.08)	0.66	(± 0.04)	0.90	0.78	(± 0.10)	0.73	(± 0.08)
+ Healthy	25	10.1	(± 5.0)	12.6	27.0	6.4	0.80	(± 0.07)	0.65	(± 0.03)	0.95	0.83	(± 0.09)	0.72	(± 0.08)
+ Diseased	56	8.1	(± 4.5)	10.2	11.9	6.4	0.76	(± 0.08)	0.66	(± 0.04)	0.88	0.76	(± 0.09)	0.73	(± 0.09)

Finally, we evaluated whether the observed connections between individual ASVs in one of the networks (i.e. the microbiome) were found consistently in the other (i.e. the pathobiome). However, such consistency was relatively rare (13.7%), indicating that many taxa were only co-occurring in either the apparently healthy or the diseased corals. However, we were able to show that consistency was strongly dependent on whether taxa benefited from the disease outbreaks or not (Fig. 8). Co-occurrence patterns between taxa benefiting from infections were relatively inconsistent, and only 8.1% of connections present within the ‘infection’ clusters were also present in the network of diseased tissues. In contrast, connections within the ‘survival’ clusters were highly conserved, and 50.7% of these connections were maintained in the microbiome of apparently healthy corals (Fig. 8).

## Discussion

Disease outbreaks can drastically reduce coral cover at local and regional scales [6, 27], and they are predicted to pose a major threat to corals on a global scale under future climate conditions [7]. We report here the emergence of a new coral disease in Micronesia, which shows slow progression rates but is capable of infecting a large array of species. Disease infections were associated with profound changes in coral-associated microbiomes and, in contrast to current ecological theory [24], characterised by a marked increase in the similarity of the communities.

### Similarity to other coral diseases

Grey-patch disease (GPD) shows many similarities with black band disease (BBD), arguably the most well-characterised coral disease to date [16]. BBD infections are linked to cyanobacterial taxa that degenerate coral tissue and cause a very distinct disease lesion [28]. Comparable to our results, cyanobacteria associated with BBD have been shown to penetrate coral tissue to the calciodermis in advance of physical signs of disease [29]. Further, both diseases show similarities in the taxonomy of disease-associated cyanobacteria. *Roseofilum* (the dominant cyanobacterium associated with BBD [18]) was recently shown to be closely related to *Rivularia* [30]. Likewise, seven of the 14 cyanobacteria that appeared to proliferate in GPD were classified as Rivulariaceae. These findings (taken collectively) suggest that there may be further similarities between the two diseases, for example, in infection dynamics and physiological impacts on the coral host.

Indeed, just as in BBD [16], combined results from histology, SEM and next-generation sequencing indicate that multiple microbes contributed to the degradation of various parts of the coral holobiont after the contraction of GPD. Besides cyanobacteria, loricate ciliates (which frequently accompany disease outbreaks [31, 32, 41, 42]) were also shown to penetrate coral tissue at the disease lesion. Further, fungal hyphae were observed to burrow into the coral skeleton and diseased tissue (Fig. 3d and Fig. 4c), a result highlighting the potential importance of this widely understudied group [33]. Interestingly, the two identified fungi in our study (*Cladosporium* and *Fusarium*) are well-described plant-associated fungi and pathogens in their own right [34].

However, there are some notable differences between GPD and BBD, for example, the role of sulphide and the changes in environmental conditions known to occur within the cyanobacterial mats or biofilms. Decreasing oxygen and increasing sulphide concentrations with biofilm depth are frequently observed in BBD, and sulphide accumulation has been highlighted as necessary for disease initiation [35]. While such chemical gradients are likely to be present in the GPD biofilm, no evidence was found of an increase in sulphide-loving bacteria at the lesion interface [16].

Indeed, there were only three non-cyanobacterial taxa among the dominant ASVs (> 0.125 relative frequency) that were consistently found at higher frequency in diseased compared to apparently healthy tissue. These included a *Myxococcales* (absent in AH tissue), an *Actibacterium* and a *Rhodobacteraceae*. *Myxococcales* have only recently been described in marine environments and are more commonly associated with soils [36]. The presence of such soil-associated bacteria on coral reefs is not unexpected and could be indicative of terrestrial run-off, with the potential to influence disease processes on reefs or result in novel diseases [37]. In contrast, the increase in the abundance of *Rhodobacteraceae* supports other studies which have linked these bacteria to diseases such as white syndrome [38]. However, similar to this study, there is little histological evidence to support such claims [39–41].

### Compositional homogeneity or ‘constrained chaos’ in the pathobiome

The Anna-Karenina principle states that the disturbance or breakdown of microbiomes is linked to increased dissimilarity between different communities as stochastic influences and chaotic community dynamics gain in importance [24]. We found that disease infections triggered a fourfold increase in microbial taxa richness, suggesting that a breakdown of the corals’ immune defence allows opportunistic taxa to colonise the infected tissue, supporting the Anna-Karenina principle at first sight. However, in contrast to our expectations, community similarity was significantly higher in diseased than apparently healthy tissues (Fig. 5b, c).

There are two complementary explanations for our findings. First, there might be unexpectedly high differences between the compositions of microbial communities associated with apparently healthy coral colonies. Indeed, there is increasing evidence that coral microbiomes are intricately linked with coral metabolism and have a large potential to affect the fitness and functionality of their hosts [21]. Further, coral colonies are characterised by long lifespans and structural adjustments of their microbiome constitute potential adaptation mechanisms to survive under changing micro-environmental conditions [42]. The resulting large variability of coral microbiomes with coral genotype, age, depth and wave exposure [15] may function (even in relatively small reefscapes) as mechanisms for niche diversification and increase microbial β-diversity.

However, the relatively large structural diversity of the microbiomes in healthy corals does not explain the high similarity we found among the pathobiomes in this study. Nor does it explain the similar results of ‘constrained chaos’ which have been shown in other diseases such as those affecting the Mediterranean coral *Cladocora caespitose* [43]. Such high similarity of the microbial communities in infected corals (i.e. compositional homogeneity of the pathobiome) implies that the host’s organisation of its own microbiome (reflected in recurrent responses to environmental conditions [44]) was replaced by other strong structuring forces. A possible mechanism explaining this phenomenon in the context of GPD is its relatively low lethality. In contrast to a number of other coral diseases (such as BBD, which can devastate whole colonies within weeks or even days [16–18]), GPD manifests relatively slow progression rates. At times, coral tissue would even grow back over the cyanobacterial biofilm. Hence, the coral holobiont and the pathobiome represent two alternative community configurations with similar competitiveness. In order to withstand the competitive pressure, certain combinations of pathogenic taxa might therefore be required to overcome the coral’s organisation of its microbiome, turning the competition between alternative community states into a structuring force homogenising pathobiomes.

Support for this hypothesis comes from the network analysis of the microbiome and the pathobiome. Connections between ASVs in the pathobiome were predominantly positive (Table 1). Moreover, we found that ASVs benefitting from infections formed apparent infection clusters of positively co-occurring taxa in the microbiomes of apparently healthy corals. However, such positive connections in correlation-based networks do have to be interpreted with care [45]. For example, such connections do not necessarily imply an interaction between species but can also represent a common response to particular environmental conditions. In the case of GPD, positive connections probably represent (at least to some degree) synergistic relationships, yet they are also equally likely to reflect a common response to a weakened immune defence of the coral colony. Regardless, both explanations imply that a parallel effort of multiple agents is required for the success of the disease, effectively increasing community similarity and ‘constraining chaos’ in GPD’s pathobiomes.

Although the above represent the most likely scenarios of what is happening when a coral contracts GPD, we cannot rule out the impact of the sampling design—especially with regard to the interpretation of the results for the diseased tissue. Our samples consisted of equal amounts of apparently healthy coral tissue (directly adjacent to the disease lesion interface) and the cyanobacterial biofilm (spreading over the denuded skeleton). We sampled in this way, as we hypothesised that the biofilm (and therefore the individual cyanobacterial species) was influential in the disease. However, assessment of the tissue without the biofilm in close proximity of the disease lesion would have been beneficial. Such an additional sample set would have allowed identification of any specific cyanobacteria singularly involved in disease onset and tissue progression, separating those which are simply free-living and non-pathogenic. We recommend this to be factored into further studies on GPD and other coral diseases exhibiting similar signs.

### Consistency of connections

The establishment of separate networks for diseased and apparently healthy corals allowed us to investigate whether connections between the same ASV were consistent in both networks. We recorded a relatively low overall consistency of 15%, a result likely explained by a number of factors. First, the reliability of network analyses is strongly dependent on sample size [46], and correlation-based assessments may fail to detect a certain percentage of connections. Further, interactions between species (represented by connections) can be strongly non-linear [47] or change with environmental conditions [48]. Both of which would lead to structural changes in the networks. We also investigated connection consistency between ASVs present in survival and infection clusters and interestingly found opposite patterns. A high prevalence (> 50%) of connections among ASVs with higher relative densities in apparently healthy corals (Fig. 8) suggests that these connections might represent stable synergistic interactions between the ASVs. According to recent network theory, such interactions can help to increase the stability of communities or species clusters [49, 50] and therefore may contribute to the persistence of these ASVs even after disease infections. Low consistency of connections in infection clusters, however, suggests that most of these ASVs are not truly positively interacting but rather jointly benefiting from certain changes within the coral holobiont. If this hypothesis proves to be correct, apparently healthy tissue (which was taken from infected corals) might already show the first ‘signs’ of infections, in a similar way to community changes that precede periodontal disease [51]. Although this is not the first time variation in the microbiome of apparently healthy tissue has been highlighted [52], it would be the first time where such infection clusters have been identified. If these are a generic component of the progression of diseases in corals, they could represent a measurable early warning sign of infection on otherwise macroscopically healthy coral colonies.

## Conclusion

In this study, we describe the grey-patch disease, a new poly-microbial coral disease, found throughout Micronesia. With a relatively slow progression rate and a generally low disease prevalence, GPD has a wide host range of common and frequently dominating coral species. One of our key findings is that multiple agents appear to be involved in degrading the corals’ defences causing the onset of this disease. In particular, we illustrate the role cyanobacteria play and infer similarities of GPD to that of black band disease. Our network analyses demonstrate the complexity of microbe-microbe interactions and reveal the presence of taxa clusters in both apparently healthy and infected corals. A further examination of these clusters represents a promising approach to increase our insight in infection dynamics and might eventually even allow us to develop tools for detecting early warning signs of disease outbreaks and/or target the microbes associated in the survival clusters for use as probiotics in coral reef restoration.

## Methods

### Disease prevalence surveys, lesion progression monitoring and sampling

Between 2011 and 2018, disease transects were conducted at multiple locations in the Indian and Pacific Oceans. Coral disease transects were conducted in the Northern Marianas, the Philippines, Chuuk, Palau, the Maldives and Reunion. Three 20 × 1-m belt transects were conducted per site (see below), and all coral colonies within the belt were assessed. Colonies were binned into pre-established size classes (currently in use in disease prevalence surveys, see [53]), identified to genus and visually inspected for signs of disease.

In the Northern Marianas, we surveyed 47 sites around 4 islands. Fifteen forereef sites and 4 shallow reef areas were surveyed in Guam between October and November 2015. Ten shallow forereef sites in Tinian, 16 shallow reef sites in Rota and 2 shallow reef flat sites in Saipan were all surveyed in May 2014. In the Philippines, 18 shallow forereef sites were surveyed in June 2011 and April 2015 including 9 at Bantayan Island (Central Visayas), 4 at Oslob, Cebu (Central Visayas), and 5 at Tubbataha. Eight shallow forereef sites on 5 islands in Chuuk were surveyed in July 2013, and 1 site was surveyed in Palau (May 2018), the Maldives (September 2017) and Reunion (March 2018). During each survey, the presence (or absence) of the newly encountered disease was assessed to gain an understanding of the possible spread across spatial scales over the specific survey periods.

At the Luminao reef flat in Guam (during November 2016), 12 colonies of *Porites lobata* were tagged (each with multiple apparently progressive lesions) and disease progression rates monitored over a 2-year period (2016–2017). The Luminao reef flat was the site of the first observations of GPD, and disease prevalence was high at this location. Monitored lesions were photographed during each census, and a 10-cm^2^ area of the lesion interface (5 cm either side of the tag) was examined using ImageJ® software [54] and assessed according to interaction type (active, stagnant, re-sheeting of coral tissue) between the GPD biofilm and the coral tissue.

Samples of the disease lesion for microscopic analysis, histology, scanning electron microscopy (SEM), 16S rRNA gene and ITS sequencing were collected during November 2016 from tagged colonies of *Porites* showing clear signs of progressive lesions (11 of the 12). From each coral, two samples were taken: one directly from the disease lesion and one from apparently healthy tissue with a minimum surface distance of 100 cm from any lesion. Sampling was performed with a sterile hammer and chisel, and each sample was immediately transported in an individual sterile zip lock bag to the University of Guam Marine Laboratory for further processing. Samples destined for histology (*n* = 22) were preserved in Z-fix; for SEM (*n* = 22), samples were preserved in glutaraldehyde; and those for 16S and ITS sequencing (*n* = 22) were preserved in 100% molecular grade ethanol and frozen immediately at − 80 °C. For 16S and ITS sequencing, three samples of the water column were collected by filtering 1 L of the water through 0.22 μm sterivex filters—see [55]. For light microscopy, 11 ‘live’ samples were collected from disease lesions and were assessed within 1 h of collection. Identification of the dominant cyanobacteria was conducted by the authors (MS and AB) and verified by experts from the light micrographs taken (Chris Lobban, Guam University, and Michael Schagerl, University of Vienna).

### Sample processing

*SEM* samples were dehydrated using 25, 50 and 70% ethanol (30 min each), followed by two 1 h baths in 100% ethanol and a final 1 h air-drying. Samples were mounted on an aluminium stub with Achesons Silver Dag (dried overnight) and coated with gold (standard 15 nm) using an Emi Tech K550X sputter coater Unit. Specimens were examined using a Stereoscan 240 scanning electron microscope, and digital images were collected by Orion 6.60.6 software.

*Histology* samples were decalcified and embedded in paraffin wax following [42]. Survey sections were cut to a thickness of 1 μm. Sections were stained with haematoxylin and eosin and investigated with a Leica DMRB light microscope.

DNA samples for 16S and ITS analysis (targeting prokaryotes and fungi, respectively) were extracted using the QIAGEN DNeasy Blood and Tissue Kit following the manufacturer’s protocols. 16S rRNA PCR amplification included a two-step process with a target region (V3–4) amplification using the universal bacterial primers MiCSQ_343FL (5′-TATGGTAATTGTCTCCTACTTRRSGCAGCAG-3′) and MiCSQ_806R (5′-AGTCAGTCAGCCGGACTACNVGGGTWTCTAAT-3′), followed by addition of indices and adaptors. Quality control and size selection were performed on the pooled amplicon sample, and then, pair-end sequencing (2 × 250 bp) of the V3–4 region was targeted with Illumina MiSeq platform [56]. For ITS analysis, denaturing gradient gel electrophoresis was conducted to attain longer fragments as a lower diversity was expected than in 16S samples. Protocols and analysis followed that outlined by [57]. In brief, the primers ITS1F (5′-CTTGGTCATTTAGAGGAAGTAA-3′) and ITS4F (5′-TCCTCCGCTTATTGATATGC-3′) were run initially, followed by a 1∶100 dilution of a second PCR product with the primers ITS3F (5′-GCATCGATGAAGAACGCAGC-3′) and ITS4F-GC (5′CGCCCGCCGCGCCCCGCGCCCGGCCCGCCG CCCCCGCCCCTCCTCCGCTTATTGATATGC-3′). Resulting bands were excised, reamplified with the latter set of ITS primers and Sanger sequenced. Contamination controls, consisting of pure ethanol and extracted in the same way as the samples, were run for both 16S and ITS sequencing.

### 16S rRNA sequence quality control and statistical analyses

Sequences were trimmed between the 10th and 190th base-pairs based on quality scores for downstream taxonomic and statistical analyses in QIIME 2 [58]. The DADA2 quality control algorithm [59] was applied to the sequencing data to retrieve the frequencies of amplicon sequence variants (ASVs). ASVs are thought to avoid taxon inflation [60] which can occur when using operational taxonomic units (OTUs)—the more commonly utilised standard for marker gene analysis. Further, ASVs assist with studies being more reproducible and comprehensive and allow for easier comparison between studies [61]. A phylogenetic tree was generated with FastTree software [62]. The taxonomic identity of ASVs was determined using the Greengenes RNA database [63] and a multinomial naive Bayesian classifier trained for the selected V4 sequence in QIIME 2. Any sequences not successfully classified by the Greengenes classifier were manually blast searched to specify taxonomy. Chloroplast sequences were excluded from downstream analyses.

Following the recommendation of Chase and Knight [64], α-biodiversity indices were calculated on absolute ASV copy numbers and on rarefied data (standardised copy number per sample). Group differences between water samples and diseased and apparently healthy coral tissues were investigated with one-way ANOVAs after data transformations to achieve homoscedacity. β-biodiversity (similarity between communities) was assessed using similarity indices (Jaccard and weighted UniFrac) and a non-metric multi-dimensional scaling (NMDS) approach. Data were converted to relative abundances prior to analysis in order to optimise the detection of group differences [65]. A PERMANOVA with Holm-adjusted *p* values (for multiple pairwise comparisons) was used to detect significant differences between groups. Systematic differences in group dispersion (variation) were examined after computing community similarity indices for all possible pairwise comparisons (using both, Jaccard and UniFrac similarity in two separated analyses) and then assessing the difference in community similarity within and between groups with a non-parametric Kruskal-Wallis test.

Further, we analysed differences in the relative densities of ASVs between apparently healthy and diseased tissues. We performed (for every ASV in our dataset) a Wilcoxon signed-rank sum test to assess whether relative abundances of individual ASVs differed between apparently healthy and infected corals. We also summed the relative frequency of all ASVs, which showed significant differences, by phylum to determine the taxonomic affiliations of ASVs with significant abundance changes. Patterns of pairwise correlations between abundances of single ASVs were assessed using a network approach [66]. We included all taxa with a mean relative abundance (across all coral tissue samples) of > 0.125% and only displayed significant correlations in our network visualisations. We repeated these processes in network analysis displaying ASV co-occurrence in apparently healthy and infected corals. Network statistics (mean degree, saturation, average connection strength) were calculated for all networks. Finally, we calculated local cluster coefficients, which indicate how well the neighbourhood of a node (in our case ASV) is interconnected [67]. It should be noted, however, that we modified computation of local clustering coefficients by multiplying the realised degree of nodes with their local clustering coefficients to account for the fact that nodes with a high degree tend to have systematically lower cluster coefficients in real-world networks [68]. All statistical analyses were performed with the statistical platform R using qgraph and HiveR packages for network visualisation [69].

## Additional file

**Additional file 1: Figure S1.** More examples of the novel coral disease named here as ‘grey patch disease’. Arrow in D indicates characteristic presence of bubbles emerging from the biofilm. **Figure S2.** Examples of the different species and genera identified as being susceptible to ‘grey patch disease’. **Figure S3.** Lesion border dynamics observed at three census periods in four lesions on a single colony. **Table S1.** All coral species observed to be susceptible to grey-patch disease during the survey period. **Table S2.** Biofilm-coral border interaction types observed at each census period. **Table S3.** Average number of sequence reads and α-diversity indices of ASVs associated with water (*n* = 3), healthy coral (*n* = 11) and diseased coral samples (*n* = 11). **Figure S4.** A modified version of Fig. 7 shown in the manuscript highlighting interactions between dominant ASVs in the microbiome of apparently healthy (above) and diseased coral tissues (below) depicted in a network representation. Lines reflect significantly positive (black) and negative (red) interactions. **Table S4.** Highlights the closest taxonomic match of the dominant bacteria identified with BLAST.

## Data Availability

All raw sequence data has been submitted to Genbank under STUDY: PRJNA448821 (SRP137688)—samples range from SRS3127585 to SRS3127608.
