# Supplementary material for: Compositional homogeneity in the pathobiome of a new, slow-spreading coral disease
Source: Microbiome. 2019 Nov 22;7:139. doi: 10.1186/s40168-019-0759-6 (PMC6873542; doi:10.1186/s40168-019-0759-6)
Supplement: Supplementary file 1 — Additional file 1: Figure S1. More examples of the novel coral disease named here as ‘grey patch disease’. Arrow in D indicates characteristic presence of bubbles emerging from the biofilm. Figure S2. Examples of the different species and genera identified as being susceptible to ‘grey patch disease’. Figure S3. Lesion border dynamics observed at three census periods in four lesions on a single colony. Table S1. All coral species observed to be susceptible to grey-patch disease during the survey period. Table S2. Biofilm-coral border interaction types observed at each census period. Table S3. Average number of sequence reads and α-diversity indices of ASVs associated with water (n = 3), healthy coral (n = 11) and diseased coral samples (n = 11). Figure S4. A modified version of Fig. 7 shown in the manuscript highlighting interactions between dominant ASVs in the microbiome of apparently healthy (above) and diseased coral tissues (below) depicted in a network representation. Lines reflect significantly positive (black) and negative (red) interactions. Table S4. Highlights the closest taxonomic match of the dominant bacteria identified with BLAST. [file 40168_2019_759_MOESM1_ESM.docx]

Supplementary Material

**
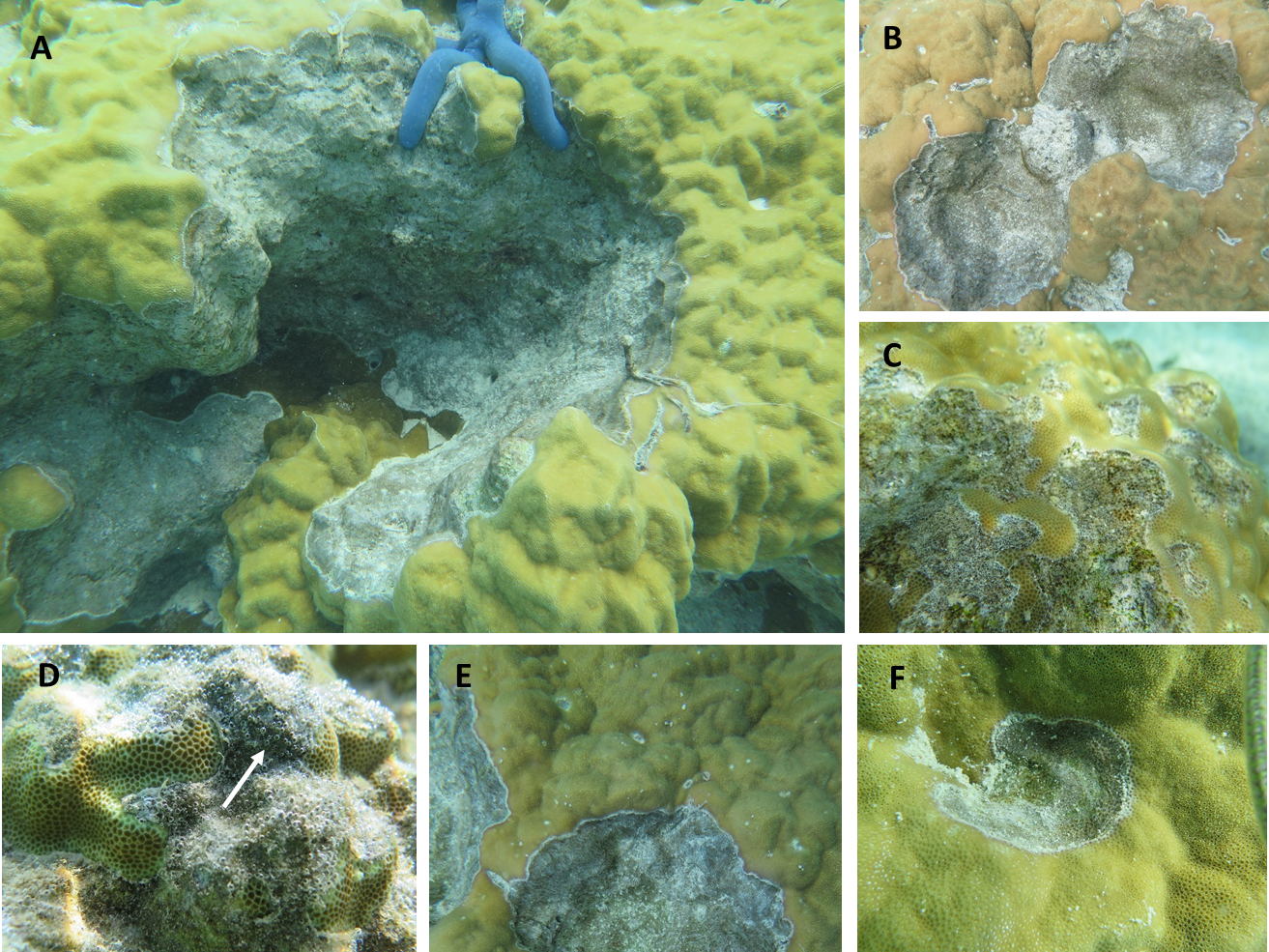
**

**Figure S1:** More examples of the novel coral disease named here as ‘grey patch disease’. Arrow in D indicates characteristic presence of bubbles emerging from the biofilm.


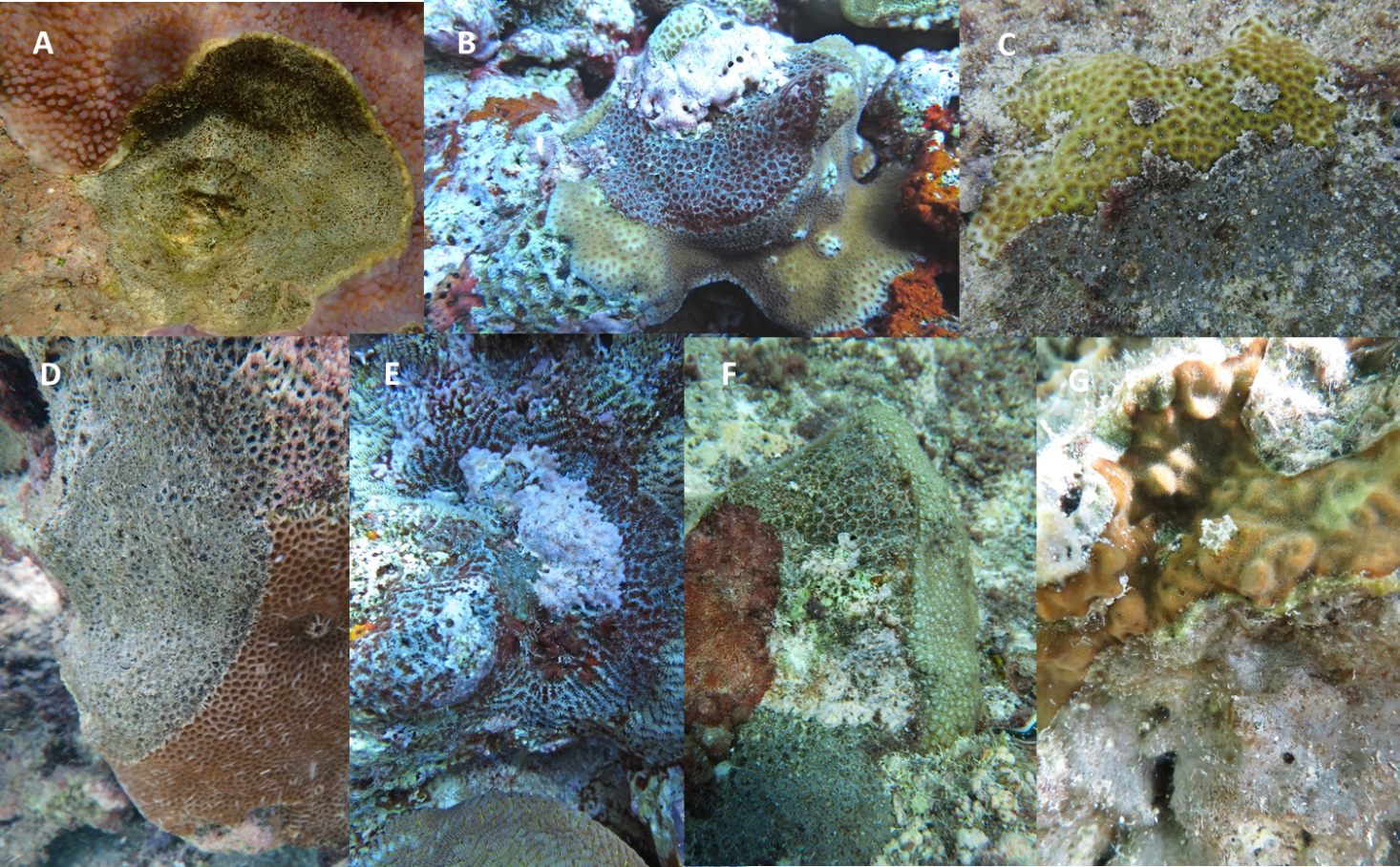


**Figure S2:** Examples of the different species and genera identified as being susceptible to ‘grey patch disease’.A = *Acropora palmarae*, B = *Goniastrea stelligera*, C = *Leptastrea purpurea*, D = *Goniastrea retriformis*,E = *Platgyra pini*, F = *Leptastrea transversa*, G = *Psammocora contigua*


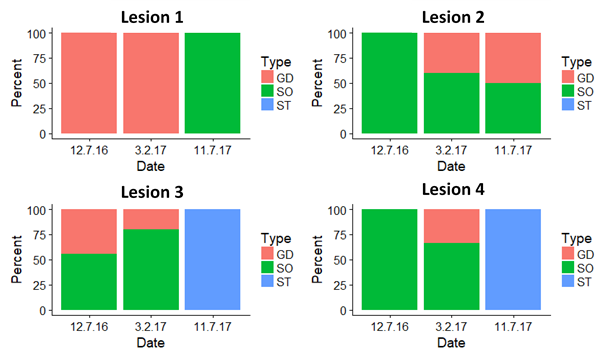


**Figure S3:** Lesion border dynamics observed at three census periods in four lesions on a single colony. GD = Grey Death progressing; SO = active stand-off, with hyperpigmentation by the coral host; ST = stasis, with no visible progression or re-sheeting, and no hyperpigmentation.

**Table S1**: All coral species observed to be susceptible to grey-patch disease during the survey period.

| **Coral species observed to show signs of grey patch disease from 2011-2018** |
| --- |
| *Porites lobate* |
| *Porites australiensis* |
| *Porites lutea* |
| *Porites rus* |
| *Porites vaughani* |
| *Leptastrea purpurea* |
| *Leptastrea transversa* |
| *Goniastrea retiformis* |
| *Acropora palmarae* |
| *Astreopora myriophthalma* |
| *Asteropora elliptica* |
| *Favia heliantoides* |
| *Montipora cf. acanthella* |
| Unidentified *Montipora* sp. |
| *Pavona frondifera* |
| *Psammacora contigua* |
| Unidentified *Montastrea* sp. |

**Table S2:** Biofilm-coral border interaction types observed at each census period. ‘No. of lesions’ refers to the number of lesions exhibiting a particular interaction type; ‘Mean % of border’ refers to the mean (+/- SD) percent of a 10-cm length of monitored border that was exhibiting that type of interaction.

| Interaction Type | 1st Census | | 2nd Census | | 3rd Census | |
| --- | --- | --- | --- | --- | --- | --- |
|  | No. of lesions | Mean % of border | No. of lesions | Mean % of border | No. of lesions | Mean % of border |
| Recovering | 1 | 65.0 | 2 | 87.5 (17.7) | 0 | 0.0 |
| Progressing | 5 | 60.8 (36.2) | 7 | 61.5 (34.9) | 3 | 36.7 (15.3) |
| Stand-Off | 7 | 85.1 (19.2) | 8 | 54.8 (22.9) | 6 | 69.0 (29.7) |
| Static | 4 | 90.0 (14.1) | 5 | 92.0 (13.0) | 7 | 96.0 (35.2) |

**Table S3**: Average number of sequence reads and α-diversity indices of ASVs associated with water (*n* = 3), healthy coral (*n* = 11) and diseased coral samples (*n* = 11). Letters denote significant differences between groups (one-way ANOVA). Data are based on absolute sequence reads and rarefaction of data did not lead to substantial changes, as total number of reads per sample were very similar between groups.

| Treatment | Species richness | | Average no. of reads ASV^-1^ | | Shannon index | | Pielou's evenness | | Simpsons index | | Hurlberts effective number of species | |
| --- | --- | --- | --- | --- | --- | --- | --- | --- | --- | --- | --- | --- |
| Diseased coral | 1295 | (±400)^a^ | 212 | (±97)^a^ | 4.5 | (±0.8)^ab^ | 0.63 | (±0.10)^a^ | 0.88 | (±0.11)^a^ | 23 | (±22)^a^ |
| Healthy coral | 333 | (±231)^b^ | 991 | (±569)^b^ | 3.5 | (±1.1)^a^ | 0.63 | (±0.23)^ab^ | 0.83 | (±0.21)^a^ | 17 | (±13)^a^ |
| Water sample | 493 | (±136)^b^ | 783 | (±126)^b^ | 5.1 | (±0.3)^b^ | 0.83 | (±0.01)^b^ | 0.97 | (±0.03)^a^ | 41 | (±14)^a^ |


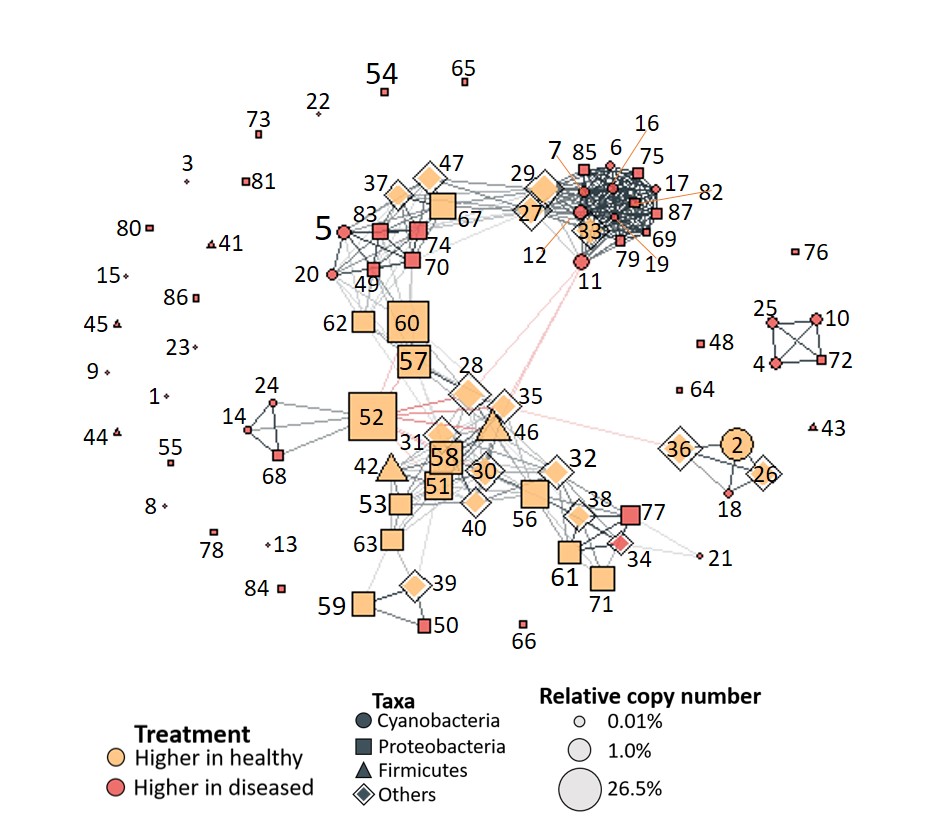


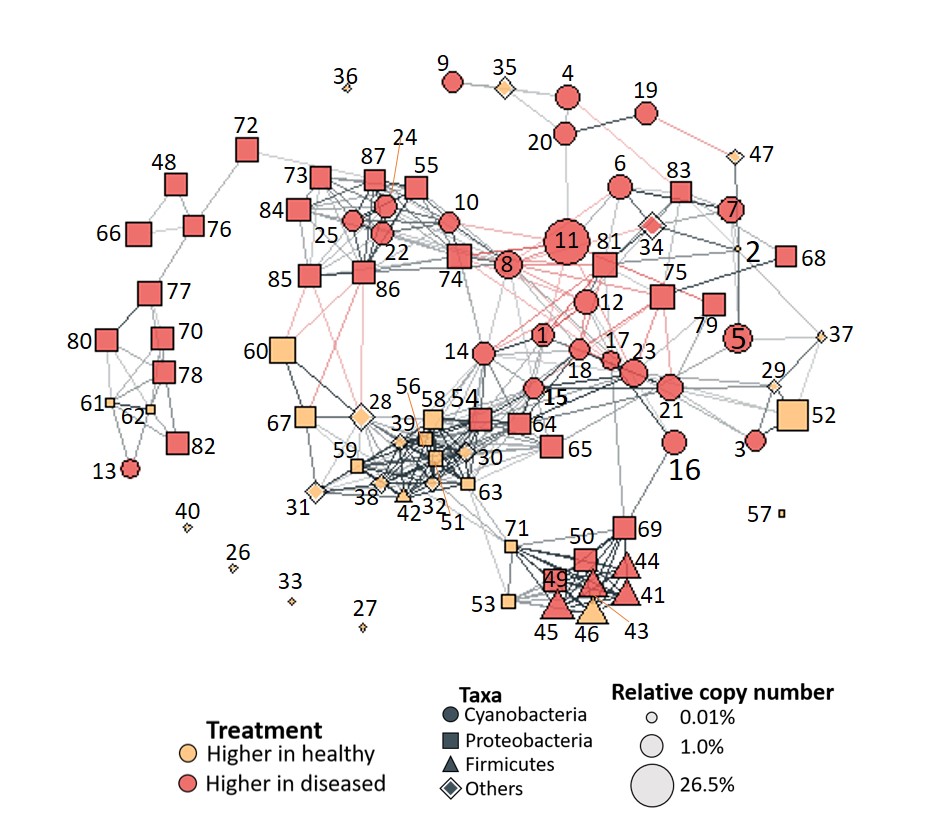


**Figure S4.** A modified version of Figure 7 shown in the manuscript highlighting Interactions between dominant ASVs in the microbiome of apparently healthy (above) and diseased coral tissues (below) depicted in a network representation. Lines reflect significantly positive (black) and negative (red) interactions. Line thickness symbolises the strength of interactions. Yellow and red fills indicate ASVs that show significantly higher densities in apparently healthy and diseased coral tissue, respectively. Phyla association of ASVs is demonstrated by different shapes. Relative size of shapes reflects their average relative contribution to total sequence reads in the sample group. Numbers here relate to individual bacteria highlighted in the associated table below (Supplementary Table 4) and the appendix 1 csv file.

**Table S4.** Highlights the closest taxonomic match of the dominant bacteria identified with BLAST. Includes the closest GenBank Accession no. if applicable, highest reliable taxonomic match and the associated Phylum. Maps to Supplementary Figure 3. The network ID number is in the first column.

| **Network ID no.** | **ASV.ID** | **GenBank**  **Accession** | **High_res_taxon** | **Phylum** |
| --- | --- | --- | --- | --- |
| 1 | [*Lyngbya* sp.](https://blast.ncbi.nlm.nih.gov/Blast.cgi#alnHdr_555929000) | [AB863133.1](https://www.ncbi.nlm.nih.gov/nucleotide/AB863133.1?report=genbank&log$=nucltop&blast_rank=1&RID=2RGXMGMD015) | *Lynbya* | Cyanobacteria |
| 2 | cyanobacterium | NA | *Oscilliatoriales* | Cyanobacteria |
| 3 | uncultured cyanobacterium | NA | *NA* | Cyanobacteria |
| 4 | [*Calothrix* sp](https://blast.ncbi.nlm.nih.gov/Blast.cgi#alnHdr_67985423) | [DQ072920.1](https://www.ncbi.nlm.nih.gov/nucleotide/DQ072920.1?report=genbank&log$=nucltop&blast_rank=5&RID=2RGFH4F2014) | *Calothrix* | Cyanobacteria |
| 5 | [*Calothrix* sp.](https://blast.ncbi.nlm.nih.gov/Blast.cgi#alnHdr_67985423) | [DQ072920.1](https://www.ncbi.nlm.nih.gov/nucleotide/DQ072920.1?report=genbank&log$=nucltop&blast_rank=5&RID=2RJ84VF1014) | *Calothrix* | Cyanobacteria |
| 6 | [*Kyrtuthrix huatulcensis*](https://blast.ncbi.nlm.nih.gov/Blast.cgi#alnHdr_1092409279) | [KT936560.1](https://www.ncbi.nlm.nih.gov/nucleotide/KT936560.1?report=genbank&log$=nucltop&blast_rank=1&RID=2RHJBCTU014) | *Kyrtuthrix* | Cyanobacteria |
| 7 | [*Calothrix* sp.](https://blast.ncbi.nlm.nih.gov/Blast.cgi#alnHdr_89213543) | [DQ380407.1](https://www.ncbi.nlm.nih.gov/nucleotide/DQ380407.1?report=genbank&log$=nucltop&blast_rank=3&RID=2RK4S1M7015) | *Calothrix* | Cyanobacteria |
| 8 | cyanobacterium | NA | *Rivulariaceae* | Cyanobacteria |
| 9 | [*Calothrix* sp.](https://blast.ncbi.nlm.nih.gov/Blast.cgi#alnHdr_975113537) | [KT336445.1](https://www.ncbi.nlm.nih.gov/nucleotide/KT336445.1?report=genbank&log$=nucltop&blast_rank=1&RID=2RHH697W015) | *Calothrix* | Cyanobacteria |
| 10 | [*Mastigocoleus*](https://blast.ncbi.nlm.nih.gov/Blast.cgi#alnHdr_645321040) | [NR_118102.1](https://www.ncbi.nlm.nih.gov/nucleotide/NR_118102.1?report=genbank&log$=nucltop&blast_rank=2&RID=2RKHDRW8014) | *Mastigocoleus* | Cyanobacteria |
| 11 | uncultured cyanobacterium | NA | *NA* | Cyanobacteria |
| 12 | uncultured cyanobacterium | NA | *NA* | Cyanobacteria |
| 13 | [*Lyngbya* sp.](https://blast.ncbi.nlm.nih.gov/Blast.cgi#alnHdr_315269896) | [HQ419195.1](https://www.ncbi.nlm.nih.gov/nucleotide/HQ419195.1?report=genbank&log$=nucltop&blast_rank=3&RID=2RHSS5MY015) | *Lynbya* | Cyanobacteria |
| 14 | [*Symploca* sp](https://blast.ncbi.nlm.nih.gov/Blast.cgi#alnHdr_752784421) | [KP182368.1](https://www.ncbi.nlm.nih.gov/nucleotide/KP182368.1?report=genbank&log$=nucltop&blast_rank=3&RID=2RGRHREW014) | *Symploca* | Cyanobacteria |
| 15 | uncultured cyanobacterium | NA | *NA* | Cyanobacteria |
| 16 | [*Leptolyngbya* sp](https://blast.ncbi.nlm.nih.gov/Blast.cgi#alnHdr_425895340) | [JQ917568.1](https://www.ncbi.nlm.nih.gov/nucleotide/JQ917568.1?report=genbank&log$=nucltop&blast_rank=8&RID=2RHUMT43014) | *Leptolyngbya* | Cyanobacteria |
| 17 | uncultured cyanobacterium | [AM177415.1](https://www.ncbi.nlm.nih.gov/nucleotide/AM177415?report=genbank&log$=nuclalign&blast_rank=1&RID=GRVD4ZVH015) | *NA* | Cyanobacteria |
| 18 | [Chroococcales](https://blast.ncbi.nlm.nih.gov/Blast.cgi#alnHdr_552050087) | [GU368107.2](https://www.ncbi.nlm.nih.gov/nucleotide/GU368107.2?report=genbank&log$=nucltop&blast_rank=2&RID=2RK25BBT015) | *Chroococcales* | Cyanobacteria |
| 19 | uncultured bacterium | NA | *Leptolyngbya* | Cyanobacteria |
| 20 | uncultured cyanobacterium | NA | *NA* | Cyanobacteria |
| 21 | [*Okeania* sp.](https://blast.ncbi.nlm.nih.gov/Blast.cgi#alnHdr_1276317362) | [LC269286.1](https://www.ncbi.nlm.nih.gov/nucleotide/LC269286.1?report=genbank&log$=nucltop&blast_rank=2&RID=2RGU1SE9014) | *Oscillatoriaceae* | Cyanobacteria |
| 22 | [*Lyngbya* sp.](https://blast.ncbi.nlm.nih.gov/Blast.cgi#alnHdr_1278113923) | [KY953146.1](https://www.ncbi.nlm.nih.gov/nucleotide/KY953146.1?report=genbank&log$=nucltop&blast_rank=2&RID=2RHZSKHN014) | *Oscillatoriaceae* | Cyanobacteria |
| 23 | [Hormoscilla](https://blast.ncbi.nlm.nih.gov/Blast.cgi#alnHdr_615292258) | [KJ439052.1](https://www.ncbi.nlm.nih.gov/nucleotide/KJ439052.1?report=genbank&log$=nucltop&blast_rank=1&RID=2RH0VVJG015) | *Hormoscilla* | Cyanobacteria |
| 24 | [Hormoscilla](https://blast.ncbi.nlm.nih.gov/Blast.cgi#alnHdr_615292258) | [KJ439052.1](https://www.ncbi.nlm.nih.gov/nucleotide/KJ439052.1?report=genbank&log$=nucltop&blast_rank=1&RID=2RJ6369A014) | *Oscillatoriaceae* | Cyanobacteria |
| 25 | [*Oscillatoria spongeliae*](https://blast.ncbi.nlm.nih.gov/Blast.cgi#alnHdr_47933461) | [AY615504.1](https://www.ncbi.nlm.nih.gov/nucleotide/AY615504.1?report=genbank&log$=nucltop&blast_rank=2&RID=2RJT9MX5015) | *Oscillatoria* | Cyanobacteria |
| 26 | uncultured bacterium | NA | *Holophagales* | Acidobacteria |
| 27 | [*Prosthecochloris* sp.](https://blast.ncbi.nlm.nih.gov/Blast.cgi#alnHdr_1214357935) | [MF423475.1](https://www.ncbi.nlm.nih.gov/nucleotide/MF423475.1?report=genbank&log$=nucltop&blast_rank=1&RID=2RHG59PB014) | *Prosthecochloris* | Chlorobi |
| 28 | [*Cloacibacterium* sp.](https://blast.ncbi.nlm.nih.gov/Blast.cgi#alnHdr_1219551635) | [LT857971.1](https://www.ncbi.nlm.nih.gov/nucleotide/LT857971.1?report=genbank&log$=nucltop&blast_rank=10&RID=2RKXRDFB015) | *Cloacibacterium* | Bacteroidetes |
| 29 | [Bacteroidetes](https://blast.ncbi.nlm.nih.gov/Blast.cgi#alnHdr_238054985) | [FJ949440.1](https://www.ncbi.nlm.nih.gov/nucleotide/FJ949440.1?report=genbank&log$=nucltop&blast_rank=5&RID=2RGDZMGV014) | *Bacteroidetes* | Bacteroidetes |
| 30 | [Sphingobacteriales](https://blast.ncbi.nlm.nih.gov/Blast.cgi#alnHdr_345847691) | [JN541141.1](https://www.ncbi.nlm.nih.gov/nucleotide/JN541141.1?report=genbank&log$=nucltop&blast_rank=12&RID=2RJWFPD1015) | *Sphingobacteriales* | Bacteroidetes |
| 31 | [Bacteroidetes](https://blast.ncbi.nlm.nih.gov/Blast.cgi#alnHdr_15027290) | [AJ318121.1](https://www.ncbi.nlm.nih.gov/nucleotide/AJ318121.1?report=genbank&log$=nucltop&blast_rank=18&RID=2RM9HK1J014) | *Sphingobacteriales* | Bacteroidetes |
| 32 | [Sphingobacteriales](https://blast.ncbi.nlm.nih.gov/Blast.cgi#alnHdr_144600747) | [EF490634.1](https://www.ncbi.nlm.nih.gov/nucleotide/EF490634.1?report=genbank&log$=nucltop&blast_rank=25&RID=2RK0KJNM014) | *Sphingobacteriales* | Bacteroidetes |
| 33 | uncultured bacterium | NA | *NA* | Bacteroidetes |
| 34 | Flavobacteriaceae | [NR_151968.1](https://www.ncbi.nlm.nih.gov/nucleotide/NR_151968?report=genbank&log$=nuclalign&blast_rank=1&RID=GRVJ3JF7015) | *Flavobacteriaceae* | Bacteroidetes |
| 35 | [uncultured](https://blast.ncbi.nlm.nih.gov/Blast.cgi#alnHdr_698039129) | NA | *Flavobacteriaceae* | Bacteroidetes |
| 36 | [Chloroflexi](https://blast.ncbi.nlm.nih.gov/Blast.cgi#alnHdr_83834249) | [DQ329932.1](https://www.ncbi.nlm.nih.gov/nucleotide/DQ329932.1?report=genbank&log$=nucltop&blast_rank=21&RID=2RHMHGUB015) | *Chloroflexales* | Chloroflexi |
| 37 | uncultured bacterium | [KT980573.1](https://www.ncbi.nlm.nih.gov/nucleotide/KT980573?report=genbank&log$=nuclalign&blast_rank=1&RID=GRW035WV014) | *NA* | SAR406 |
| 38 | [Streptomyces](https://blast.ncbi.nlm.nih.gov/Blast.cgi#alnHdr_371909352) | [FN646678.1](https://www.ncbi.nlm.nih.gov/nucleotide/FN646678.1?report=genbank&log$=nucltop&blast_rank=1&RID=2RGYXHT5014) | *Streptomyces* | Actinobacteria |
| 39 | [Glycomyces](https://blast.ncbi.nlm.nih.gov/Blast.cgi#alnHdr_219846201) | [NR_025791.1](https://www.ncbi.nlm.nih.gov/nucleotide/NR_025791.1?report=genbank&log$=nucltop&blast_rank=1&RID=2RKNBPF6015) | *Glycomyces* | Actinobacteria |
| 40 | [Acidobacteriaceae](https://blast.ncbi.nlm.nih.gov/Blast.cgi#alnHdr_765490618) | [KP717534.1](https://www.ncbi.nlm.nih.gov/nucleotide/KP717534.1?report=genbank&log$=nucltop&blast_rank=5&RID=2RKWEXUG015) | *Acidobacteriaceae* | Acidobacteria |
| 41 | [Lactobacillus](https://blast.ncbi.nlm.nih.gov/Blast.cgi#alnHdr_1279336818) | [MG551162.1](https://www.ncbi.nlm.nih.gov/nucleotide/MG551162.1?report=genbank&log$=nucltop&blast_rank=2&RID=2RK76HDR014) | *Lactobacillus* | Firmicutes |
| 42 | [*Streptococcus* sp](https://blast.ncbi.nlm.nih.gov/Blast.cgi#alnHdr_1274590840) | [KY321509.1](https://www.ncbi.nlm.nih.gov/nucleotide/KY321509.1?report=genbank&log$=nucltop&blast_rank=1&RID=2RGW6FDT015) | *Streptococcus* | Firmicutes |
| 43 | [*Listeria monocytogenes*](https://blast.ncbi.nlm.nih.gov/Blast.cgi#alnHdr_1282991706) | [CP023321.1](https://www.ncbi.nlm.nih.gov/nucleotide/CP023321.1?report=genbank&log$=nucltop&blast_rank=1&RID=2RG4HVPC015) | [*Listeria monocytogenes*](https://blast.ncbi.nlm.nih.gov/Blast.cgi#alnHdr_1282991706) | Firmicutes |
| 44 | [Enterococcus](https://blast.ncbi.nlm.nih.gov/Blast.cgi#alnHdr_1280040211) | MG557615.1 | *Enterococcus* | Firmicutes |
| 45 | [*Bacillus* sp](https://blast.ncbi.nlm.nih.gov/Blast.cgi#alnHdr_1280039174) | [KY923226.1](https://www.ncbi.nlm.nih.gov/nucleotide/KY923226.1?report=genbank&log$=nucltop&blast_rank=1&RID=2RKRCREG015) | *Bacillus* | Firmicutes |
| 46 | [Staphylococcus](https://blast.ncbi.nlm.nih.gov/Blast.cgi#alnHdr_1280072350) | [MG575995.1](https://www.ncbi.nlm.nih.gov/nucleotide/MG575995.1?report=genbank&log$=nucltop&blast_rank=1&RID=2RGJ78RD015) | *Staphylococcus* | Firmicutes |
| 47 | [Spirochaetes](https://blast.ncbi.nlm.nih.gov/Blast.cgi#alnHdr_300714171) | [GQ484168.1](https://www.ncbi.nlm.nih.gov/nucleotide/GQ484168.1?report=genbank&log$=nucltop&blast_rank=5&RID=2RH3XYKB015) | *Spirochaetales* | Spirochaetes |
| 48 | [*Desulfovibrio* sp.](https://blast.ncbi.nlm.nih.gov/Blast.cgi#alnHdr_284021947) | [GU299787.1](https://www.ncbi.nlm.nih.gov/nucleotide/GU299787.1?report=genbank&log$=nucltop&blast_rank=33&RID=2RJRKFCK015) | *Desulfovibrio* | Proteobacteria |
| 49 | [Salmonella](https://blast.ncbi.nlm.nih.gov/Blast.cgi#alnHdr_1279395547) | [KY776588.1](https://www.ncbi.nlm.nih.gov/nucleotide/KY776588.1?report=genbank&log$=nucltop&blast_rank=1&RID=2RGSSUKA015) | *Salmonella* | Proteobacteria |
| 50 | [*Shigella*](https://blast.ncbi.nlm.nih.gov/Blast.cgi#alnHdr_1280072393) | [MG576038.1](https://www.ncbi.nlm.nih.gov/nucleotide/MG576038.1?report=genbank&log$=nucltop&blast_rank=1&RID=2RGP5N7N014) | *Shigella* | Proteobacteria |
| 51 | [*Aeromonas*](https://blast.ncbi.nlm.nih.gov/Blast.cgi#alnHdr_1280072384) | [MG576029.1](https://www.ncbi.nlm.nih.gov/nucleotide/MG576029.1?report=genbank&log$=nucltop&blast_rank=1&RID=2RJV0WNM015) | *Aeromonas* | Proteobacteria |
| 52 | [Uncultured gamma proteobacterium](https://blast.ncbi.nlm.nih.gov/Blast.cgi#alnHdr_149208999) | NA | *Oceanospirillales* | Proteobacteria |
| 53 | *Endozoicomonas* | [LN875493.1](https://www.ncbi.nlm.nih.gov/nucleotide/LN875493?report=genbank&log$=nuclalign&blast_rank=1&RID=GRWC38MK014) | *Endozoicomonas* | Proteobacteria |
| 54 | uncultured bacterium | NA | *NA* | Proteobacteria |
| 55 | [*Haliea* sp.](https://blast.ncbi.nlm.nih.gov/Blast.cgi#alnHdr_383502318) | [AB646260.1](https://www.ncbi.nlm.nih.gov/nucleotide/AB646260.1?report=genbank&log$=nucltop&blast_rank=9&RID=2RK9S13E015) | *Haliea* | Proteobacteria |
| 56 | [Acidovorax](https://blast.ncbi.nlm.nih.gov/Blast.cgi#alnHdr_1280072372) | [MG576017.1](https://www.ncbi.nlm.nih.gov/nucleotide/MG576017.1?report=genbank&log$=nucltop&blast_rank=2&RID=2RMB2TXE014) | *Acidovorax* | Proteobacteria |
| 57 | [Brachymonas](https://blast.ncbi.nlm.nih.gov/Blast.cgi#alnHdr_1274080567) | [MG231211.1](https://www.ncbi.nlm.nih.gov/nucleotide/MG231211.1?report=genbank&log$=nucltop&blast_rank=1&RID=2RJFG30G015) | *Comamonadaceae* | Proteobacteria |
| 58 | [*Pelomonas* sp.](https://blast.ncbi.nlm.nih.gov/Blast.cgi#alnHdr_1252310772) | [MG052169.1](https://www.ncbi.nlm.nih.gov/nucleotide/MG052169.1?report=genbank&log$=nucltop&blast_rank=2&RID=2RH9NTCJ015) | *Pelomonas* | Proteobacteria |
| 59 | [*Metallibacterium* sp.](https://blast.ncbi.nlm.nih.gov/Blast.cgi#alnHdr_695102056) | [KM108685.1](https://www.ncbi.nlm.nih.gov/nucleotide/KM108685.1?report=genbank&log$=nucltop&blast_rank=21&RID=2RMHEYMB014) | *Metallibacterium* | Proteobacteria |
| 60 | [*Acinetobacter*](https://blast.ncbi.nlm.nih.gov/Blast.cgi#alnHdr_1277450994) sp. | [MG517433.1](https://www.ncbi.nlm.nih.gov/nucleotide/MG517433.1?report=genbank&log$=nucltop&blast_rank=1&RID=2RJM496F015) | *Acinetobacter* | Proteobacteria |
| 61 | [*Acinetobacter* sp.](https://blast.ncbi.nlm.nih.gov/Blast.cgi#alnHdr_1279489537) | [LC333628.1](https://www.ncbi.nlm.nih.gov/nucleotide/LC333628.1?report=genbank&log$=nucltop&blast_rank=1&RID=2RK66HXY014) | *Acinetobacter* | Proteobacteria |
| 62 | [*Acinetobacter*](https://blast.ncbi.nlm.nih.gov/Blast.cgi#alnHdr_1280039173) sp. | [KY930369.1](https://www.ncbi.nlm.nih.gov/nucleotide/KY930369.1?report=genbank&log$=nucltop&blast_rank=1&RID=2RJ4BU7B015) | *Acinetobacter* | Proteobacteria |
| 63 | [Moraxella](https://blast.ncbi.nlm.nih.gov/Blast.cgi#alnHdr_1282077041) | [CP024185.2](https://www.ncbi.nlm.nih.gov/nucleotide/CP024185.2?report=genbank&log$=nucltop&blast_rank=1&RID=2RJJ7ASH014) | *Moraxella* | Proteobacteria |
| 64 | [Sorangiineae](https://blast.ncbi.nlm.nih.gov/Blast.cgi#alnHdr_404427961) | [JQ515607.1](https://www.ncbi.nlm.nih.gov/nucleotide/JQ515607.1?report=genbank&log$=nucltop&blast_rank=20&RID=2RKDTU8C014) | *Sorangiineae* | Proteobacteria |
| 65 | [Myxococcales](https://blast.ncbi.nlm.nih.gov/Blast.cgi#alnHdr_330374356) | [JF413339.1](https://www.ncbi.nlm.nih.gov/nucleotide/JF413339.1?report=genbank&log$=nucltop&blast_rank=8&RID=2RKSM4RW015) | *Myxococcales* | Proteobacteria |
| 66 | [Myxococcales](https://blast.ncbi.nlm.nih.gov/Blast.cgi#alnHdr_330374356) | [JF413339.1](https://www.ncbi.nlm.nih.gov/nucleotide/JF413339.1?report=genbank&log$=nucltop&blast_rank=37&RID=2RGM0G0Z015) | *Myxococcales* | Proteobacteria |
| 67 | [Desulfobacteraceae](https://blast.ncbi.nlm.nih.gov/Blast.cgi#alnHdr_411115982) | [KC009880.1](https://www.ncbi.nlm.nih.gov/nucleotide/KC009880.1?report=genbank&log$=nucltop&blast_rank=17&RID=2RHXWY68014) | *Desulfobacteraceae* | Proteobacteria |
| 68 | uncultured bacterium | [KT976710.1](https://www.ncbi.nlm.nih.gov/nucleotide/KT976710?report=genbank&log$=nuclalign&blast_rank=1&RID=GRWFS62X015) | *NA* | Proteobacteria |
| 69 | [*Rhodospirillum* sp.](https://blast.ncbi.nlm.nih.gov/Blast.cgi#alnHdr_321172600) | [HQ877494.1](https://www.ncbi.nlm.nih.gov/nucleotide/HQ877494.1?report=genbank&log$=nucltop&blast_rank=10&RID=2RM5HSRA015) | *Rhodospirillum* | Proteobacteria |
| 70 | [*Erythrobacter* sp](https://blast.ncbi.nlm.nih.gov/Blast.cgi#alnHdr_1245699176) | [KX989418.1](https://www.ncbi.nlm.nih.gov/nucleotide/KX989418.1?report=genbank&log$=nucltop&blast_rank=1&RID=2RJY5G5D014) | *Erythrobacter* | Proteobacteria |
| 71 | [Sphingomonas](https://blast.ncbi.nlm.nih.gov/Blast.cgi#alnHdr_1253294515) | [CP023705.1](https://www.ncbi.nlm.nih.gov/nucleotide/CP023705.1?report=genbank&log$=nucltop&blast_rank=2&RID=2RKBKEVD015) | *Sphingomonas* | Proteobacteria |
| 72 | [*Boseongicola* sp](https://blast.ncbi.nlm.nih.gov/Blast.cgi#alnHdr_829488187) | [KP410674.1](https://www.ncbi.nlm.nih.gov/nucleotide/KP410674.1?report=genbank&log$=nucltop&blast_rank=10&RID=2RM33V4M015) | *Boseongicola* | Proteobacteria |
| 73 | [*Pseudoruegeria* sp.](https://blast.ncbi.nlm.nih.gov/Blast.cgi#alnHdr_1245699015) | [KX989257.1](https://www.ncbi.nlm.nih.gov/nucleotide/KX989257.1?report=genbank&log$=nucltop&blast_rank=10&RID=2RKTX6YF015) | *Pseudoruegeria* | Proteobacteria |
| 74 | [*Pseudoruegeria* sp.](https://blast.ncbi.nlm.nih.gov/Blast.cgi#alnHdr_1245699015) | [KX989257.1](https://www.ncbi.nlm.nih.gov/nucleotide/KX989257.1?report=genbank&log$=nucltop&blast_rank=2&RID=2RJDSP2E014) | *Rhodobacteraceae* | Proteobacteria |
| 75 | [*Oceanicola* sp.](https://blast.ncbi.nlm.nih.gov/Blast.cgi#alnHdr_536591893) | [KF500417.1](https://www.ncbi.nlm.nih.gov/nucleotide/KF500417.1?report=genbank&log$=nucltop&blast_rank=15&RID=2RHW6BC2015) | *Oceanicola* | Proteobacteria |
| 76 | [Rhodobacteraceae](https://blast.ncbi.nlm.nih.gov/Blast.cgi#alnHdr_219693266) | [FJ516789.1](https://www.ncbi.nlm.nih.gov/nucleotide/FJ516789.1?report=genbank&log$=nucltop&blast_rank=3&RID=2RKJP2V9015) | *Rhodobacteraceae* | Proteobacteria |
| 77 | [Actibacterium](https://blast.ncbi.nlm.nih.gov/Blast.cgi#alnHdr_566085127) | [NR_108866.1](https://www.ncbi.nlm.nih.gov/nucleotide/NR_108866.1?report=genbank&log$=nucltop&blast_rank=31&RID=2RHP3UPE014) | *Actibacterium* | Proteobacteria |
| 78 | [Pseudooceanicola](https://blast.ncbi.nlm.nih.gov/Blast.cgi#alnHdr_1278971890) | [KY273603.2](https://www.ncbi.nlm.nih.gov/nucleotide/KY273603.2?report=genbank&log$=nucltop&blast_rank=18&RID=2RK86X2D014) | *Pseudooceanicola* | Proteobacteria |
| 79 | [Rhodobacteraceae](https://blast.ncbi.nlm.nih.gov/Blast.cgi#alnHdr_343174908) | [JN232306.1](https://www.ncbi.nlm.nih.gov/nucleotide/JN232306.1?report=genbank&log$=nucltop&blast_rank=11&RID=2RKZ462N015) | *Rhodobacteraceae* | Bacteroidetes |
| 80 | [Roseovarius](https://blast.ncbi.nlm.nih.gov/Blast.cgi#alnHdr_1212783592) | [MF403134.1](https://www.ncbi.nlm.nih.gov/nucleotide/MF403134.1?report=genbank&log$=nucltop&blast_rank=1&RID=2RH7DNRG014) | *Roseovarius* | Proteobacteria |
| 81 | [*Roseovarius* sp.](https://blast.ncbi.nlm.nih.gov/Blast.cgi#alnHdr_1212783592) | [MF403134.1](https://www.ncbi.nlm.nih.gov/nucleotide/MF403134.1?report=genbank&log$=nucltop&blast_rank=10&RID=2RJ2T81E014) | *Roseovarius* | Proteobacteria |
| 82 | [Rhodobacteraceae](https://blast.ncbi.nlm.nih.gov/Blast.cgi#alnHdr_404428411) | [JQ516588.1](https://www.ncbi.nlm.nih.gov/nucleotide/JQ516588.1?report=genbank&log$=nucltop&blast_rank=3&RID=2RGAA3WE015) | *Rhodobacteraceae* | Proteobacteria |
| 83 | [*Rhodovulum* sp](https://blast.ncbi.nlm.nih.gov/Blast.cgi#alnHdr_539360348) | [KF500855.1](https://www.ncbi.nlm.nih.gov/nucleotide/KF500855.1?report=genbank&log$=nucltop&blast_rank=31&RID=2RMFW0R9014) | *Rhodovulum* | Proteobacteria |
| 84 | [Phyllobacteriaceae](https://blast.ncbi.nlm.nih.gov/Blast.cgi#alnHdr_1229620106) | [MF629007.1](https://www.ncbi.nlm.nih.gov/nucleotide/MF629007.1?report=genbank&log$=nucltop&blast_rank=1&RID=2RHB3UWU014) | *Phyllobacteriaceae* | Proteobacteria |
| 85 | [*Salinovum* sp.](https://blast.ncbi.nlm.nih.gov/Blast.cgi#alnHdr_1275552485) | [MG456786.1](https://www.ncbi.nlm.nih.gov/nucleotide/MG456786.1?report=genbank&log$=nucltop&blast_rank=1&RID=2RHETPF7014) | *Salinovum* | Proteobacteria |
| 86 | [Maritimibacter](https://blast.ncbi.nlm.nih.gov/Blast.cgi#alnHdr_631252830) | [NR_114028.1](https://www.ncbi.nlm.nih.gov/nucleotide/NR_114028.1?report=genbank&log$=nucltop&blast_rank=7&RID=2RJZ79M7014) | *Maritimibacter* | Proteobacteria |
| 87 | [*Jannaschia* sp.](https://blast.ncbi.nlm.nih.gov/Blast.cgi#alnHdr_1216722817) | [MF497080.1](https://www.ncbi.nlm.nih.gov/nucleotide/MF497080.1?report=genbank&log$=nucltop&blast_rank=1&RID=2RHCH788014) | *Jannaschia* | Proteobacteria |
